# Twofold fused concave hosts containing two phosphorus atoms: modules for the sandwich-type encapsulation of fullerenes in variable cavities†
†Electronic supplementary information (ESI) available. CCDC 1401373–1401379. For ESI and crystallographic data in CIF or other electronic format see DOI: 10.1039/c5sc02224j


**DOI:** 10.1039/c5sc02224j

**Published:** 2015-07-24

**Authors:** Masaki Yamamura, Daigo Hongo, Tatsuya Nabeshima

**Affiliations:** a Graduate School of Pure and Applied Sciences , Tsukuba Research Center for Interdisciplinary Materials Science , University of Tsukuba , 1-1-1 Tennodai , Tsukuba , Ibaraki , 305-8571 Japan . Email: myama@chem.tsukuba.ac.jp ; Email: nabesima@chem.tsukuba.ac.jp

## Abstract

The design and synthesis of extended concave host **P2** by fusion of two concave phosphorus-containing units is reported. Co-crystallization of **P2** and the fullerene guests C_60_ and C_70_ afforded the 2 : 1 host–guest complexes (**P2**)_2_ ⊃ C_60_ and (**P2**)_2_ ⊃ C_70_, in which the two concave surfaces of **P2** encapsulate the convex surface of the fullerenes in a sandwich fashion. Interestingly, the orientation of the two **P2** molecules with respect to each other was observed to be flexible, resulting in the formation of a variety of cavity shapes. MALDI-TOF mass, NMR, and UV-vis absorption spectra supported the formation of host–guest complexes between **P2** and the fullerenes in solution. The affinity of **P2**, containing two phosphorus atoms, towards fullerenes was significantly enhanced relative to **P1** with one phosphorus atom.

## Abstract 2

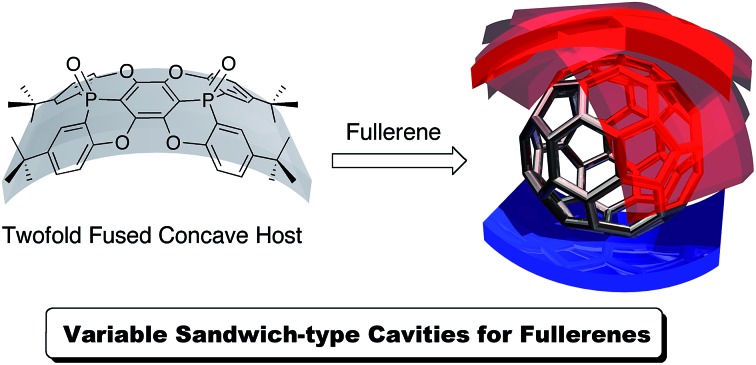
Concave host by fusion of two phosphorus atoms affords variable sandwich-type cavities for guest fullerene, C_60_ or C_70_.

## Introduction

**Chart 1:**
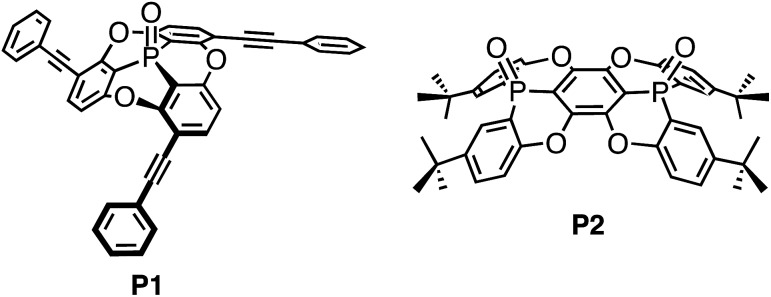
Concave host **P1** and twofold fused concave host **P2**.

The recognition of fullerenes is one of the most intensively pursued research subjects in contemporary supramolecular and host–guest chemistry.^1^ The applications of fullerene recognition are manifold and reach from fullerene purification^2^ and nanoscale organisation^3^ to the formation of photovoltaic cells.^4^ A central paradigm for the design of molecular hosts is the generation of well-defined three-dimensional architectures so that suitable arrangements of the binding sites are obtained.^5^ For the recognition and capture of fullerenes, which usually do not contain any functional groups, only weak interactions such as π–π and CH–π interactions are available, and therefore aromatic compounds have often been used as binding sites. Even though benzene rings represent a relatively small binding site, recent studies have demonstrated a very high affinity for fullerene derivatives arising from the macrocyclic arrangement of benzene rings (carbon-nanorings),^6^ and can therefore be considered as an analogy of crown ether chemistry. Coordinative self-assembly of small molecules is also effective to capture fullerenes.^7^ Concave π-conjugated molecules^8^,^9^ are also promising binding sites for fullerenes, as their concave surfaces resemble and match the convex π-surface of fullerenes.^10^,^11^ Although individual concave molecules exhibit merely a weak affinity towards fullerenes, the fusion of multiple molecular units can substantially increase this affinity.^12^,^13^ Recently, we reported phosphorus-containing concave molecule **P1** (Chart 1),^14^,^15^ and although four host molecules perfectly wrapped around the convex surface of C_60_ in the crystalline state, the host–guest interaction between the host and the C_60_ guest was found to be negligible in organic solvents. Concave **P1** is considered to be a good molecular host fragment, but a carefully crafted arrangement of multiple such fragments is necessary for an efficient recognition of C_60_.

Therefore, we have designed and synthesised the extended concave molecule **P2** by fusing two phosphorus-containing concave units similar to **P1**. The expanded concave surface of **P2** is expected to enhance the affinity of **P2** towards fullerene guests through increased concave–convex interactions.

## Results and discussion

### Synthesis of the fused concave host

**Scheme 1:**
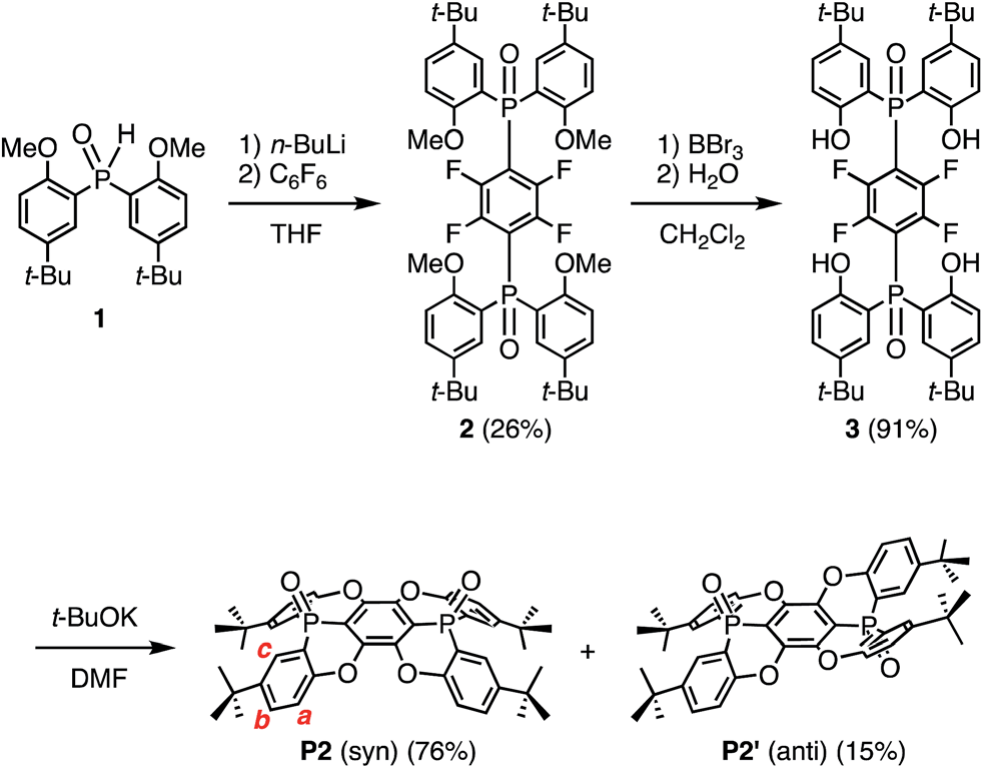
Synthesis of bowl-shaped phosphorus-containing host **P2**.

Concave host **P2** was synthesised in three steps (Scheme 1), starting from the reaction between hexafluorobenzene and two equivalents of an anion, which was generated from the deprotonation of **1** with *n*-BuLi. Thus, 1,4-bis(phosphoryl)tetrafluorobenzene **2** was obtained in 26% yield.^16^ Subsequent treatment of **2** with BBr_3_ resulted in the removal of four methyl groups to afford **3** in 91% yield. Deprotonation of **3** with *t*-BuOK furnished concave **P2** (76%) and its *anti*-isomer **P2′** (15%) in excellent stereo-selectivity, whereby the intramolecular S_N_Ar reaction is the key step. The molecular structures of **P2** and **P2′** were determined unequivocally by single crystal X-ray diffraction analysis (Fig. S27†).

**Fig. 1:**
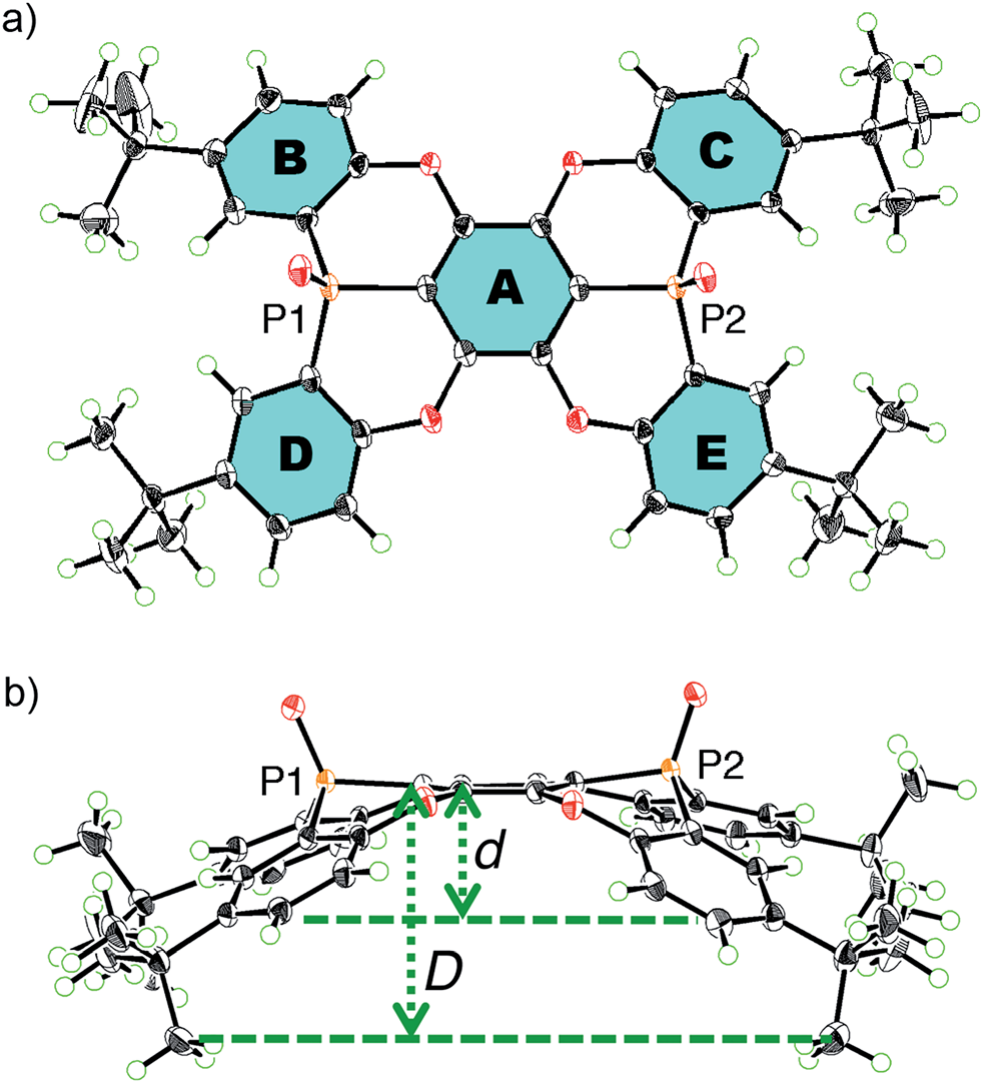
(a) Top and (b) side views of an *ORTEP* drawing of **P2** with thermal ellipsoids set at 50% probability.

Single crystals of **P2**, suitable for X-ray crystallographic analysis, were obtained by recrystallization from CHCl_3_/hexane, and the crystal structure clearly showed the concave shape of **P2** (Fig. 1). The two phosphorus atoms, P1 and P2, are positioned slightly above (0.167/0.269 Å) the plane of the central benzene ring **A**.^17^ The P–O axes are aligned almost vertically with respect to **A**, comprising dihedral O–P–C–C angles of 82.0° (P1) and 88.2° (P2). Accordingly, no mirror planes pass through the two phosphorus atoms, resulting in a molecular structure of **P2** with low symmetry. One P–O bond is twisted in clockwise direction, while the other is twisted in the opposite direction. Torsion angles of 24.2° and 17.4° were observed between the central ring **A** and the terminal **B** and **C** rings, respectively. Much larger values were observed between **A** and **D** (32.2°) or **E** (38.4°). This distorted structure is probably caused by steric repulsion between the **B**/**D** or **C**/**E** benzene rings. In contrast to the molecular structure in the crystal, the ^1^H NMR spectrum displayed four magnetically equivalent terminal benzene rings (**B–E**), most likely due to a fast interconversion of the twisting on the NMR timescale.

For extended concave hosts such as **P2**, two depth parameters can be defined in order to characterise the concave surface (Fig. S26†). The first (*d*) is defined as the distance between the centroids of **A** and the plane of the *t*-Bu-substituted carbon atoms in **B–E** (Fig. 1). The second (*D*) is defined as the distance between the centroids of **A** and the plane of the terminal methyl groups. For **P2**, depth values of 1.8 Å (*d*) and 3.6 Å (*D*) were observed, *i.e.***P2** may be considered a deep concave host, if the methyl groups are included as host fragments.

### Encapsulation of fullerenes

Three different types of black single co-crystals of **P2** and C_60_ (**I–III**), suitable for X-ray crystallographic analysis, were obtained by diffusing hexane vapour into solutions of **P2** and C_60_ in toluene/CHCl_3_, anisole, or CHCl_3_, respectively. These crystals consist of 2 : 1 host–guest complexes (**P2**)_2_ ⊃ C_60_ with centrosymmetric space groups *P*2_1_/*c* (**I**), *C*2/*m* (**II**), and *C*2/*c* (**III**). In **I–III**, the two concave surfaces of **P2** surround the convex surface of C_60_ in a sandwich fashion, and the differences between **I–III** is ascribed to different solvates.^18^ The orientation of the encapsulated C_60_ guest molecule could not be determined accurately, due to the presence of disorder. This is contrary to the previously reported tetrahedral host–guest complex between C_60_ and **P1**, for which no disorder of C_60_ within the tetrahedral cavity was observed.^14^

**Fig. 2:**
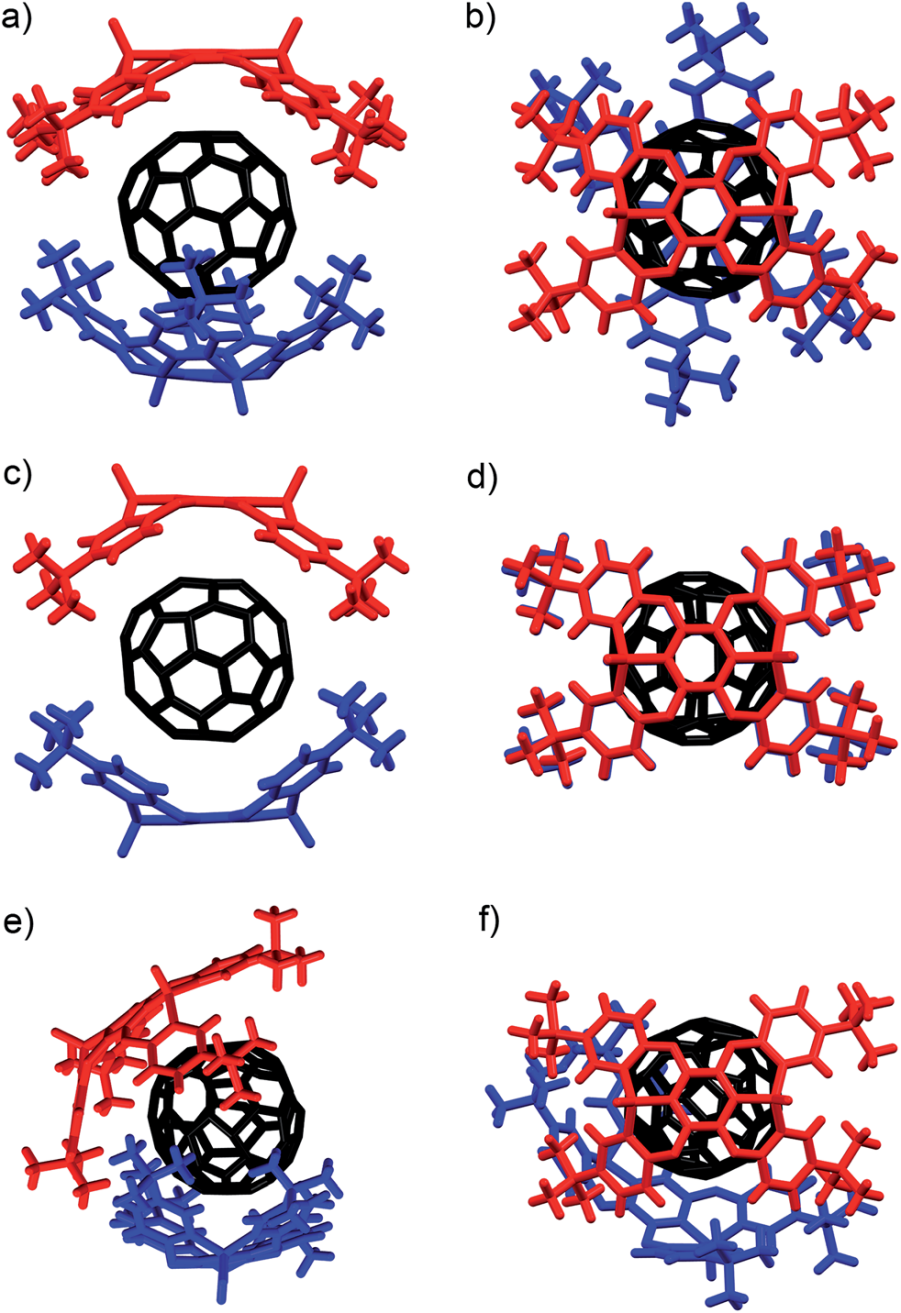
Molecular structures of the (**P2**)_2_ ⊃ C_60_ forms **I–III**. (a) Side and (b) top view of **I** (twisted); (c) side and (d) top view of **II** (horizontal); (e) side and (f) top view of **III** (misaligned).

In **I**, two host molecules encapsulated C_60_ in a sandwich fashion, *i.e.* the host molecules occupy opposing apex positions of the guest (Fig. 2a). The distance between the **A** centroids in the two hosts is 13.2 Å, which corresponds to the sum of a benzene ring and the diameter of C_60_. In contrast, distances of 6.8–7.0 Å were observed between the centroids of **B–E** and C_60_, which is longer than the sum of the van der Waals radii of C_60_ and a carbon atom (6.5 Å). The observed lengths thus suggested that the fullerene should be in direct contact with the two concave surfaces of both host molecules. The P1–P2 axes of the two host molecule are offset by 67.6° with respect to each other (Fig. 2b), reflecting the twisted arrangement of the two **P2** molecules in **I**. In **II**, two comparable, yet crystallographically independent (**P2**)_2_ ⊃ C_60_ complexes are contained within the asymmetric unit (Fig. S29 and S30†). The host molecules are arranged in a similar manner to **I**, and the distance between the **A** centroids of the two hosts is 13.2 Å (Fig. 2c). In contrast to **I**, the P1–P2 axes of the two hosts are aligned in **II** (Fig. 2d), reflecting a horizontal arrangement of the two **P2** molecules in **II**. In **III**, the two hosts do not occupy opposing apex positions of the C_60_ guest, as one host molecule is positioned at a latitudinal angle of 54.3° relative to the apex position (Fig. 2e), reflecting the misalignment of the two **P2** molecules in **III**. Based on these observations, it can be concluded that the surface of C_60_ is too large to be covered entirely by two **P2** host molecules, but simultaneously too small to be covered by three **P2** host molecules. This mismatch in size should be at least partially responsible for the formation of different crystal forms in these host–guest complexes.

**Fig. 3:**
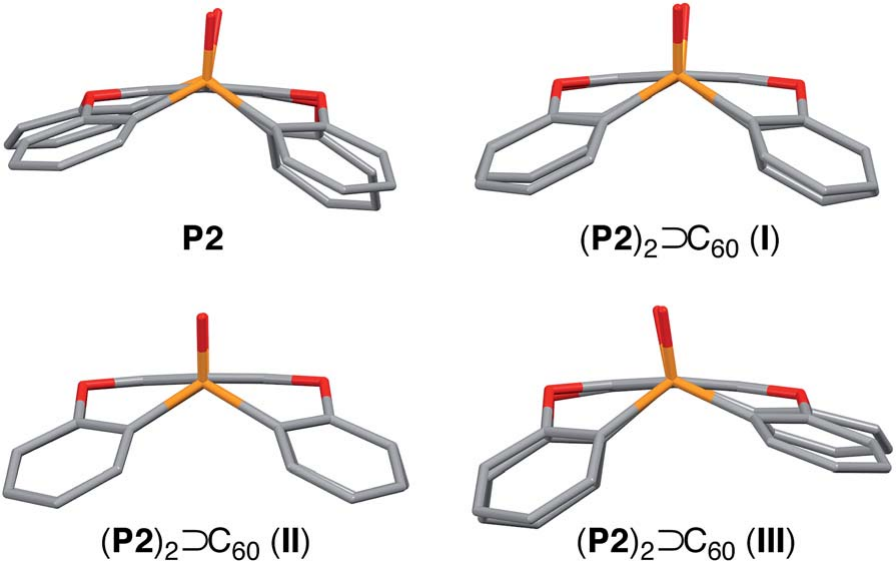
Comparison of the host **P2** moiety in uncomplexed **P2** and in **I–III**.

The internal structure of the **P2** hosts was observed to vary in the different crystals (Fig. 3). In **I**, a significantly smaller deviation of the torsion angles (29.5–37.4°) between the central (**A**) and terminal benzene rings (**B–E**) in **P2** was observed (Table 1) relative to that in uncomplexed **P2** (17.4–38.4°). While the corresponding deviation in **II** was observed to be even smaller (33.9–35.7°), that in **III** is larger (17.3–41.5°), and hence, **P2** should be able to adapt its structure according to the specific architecture of the host–guest complex.

**Table 1:** Structural parameters for the concave moiety in **P2** and **I–III**

	**P2**	(**P2**)_2_ ⊃ C_60_ (**I**)	(**P2**)_2_ ⊃ C_60_ (**II**)	(**P2**)_2_ ⊃ C_60_ (**III**)
**Bowl depth**				
*d* (Å)	1.81	2.40	2.32	2.04
*D* (Å)	3.56	4.60	4.22	4.07

**Torsion angles**				
**A–B** (°)	24.2	29.5	33.9	23.2
**A–C** (°)	17.4	33.8	35.7	17.3
**A–E** (°)	32.2	37.4	33.9	41.5
**A–F** (°)	38.4	34.4	35.7	41.0

**Fig. 4:**
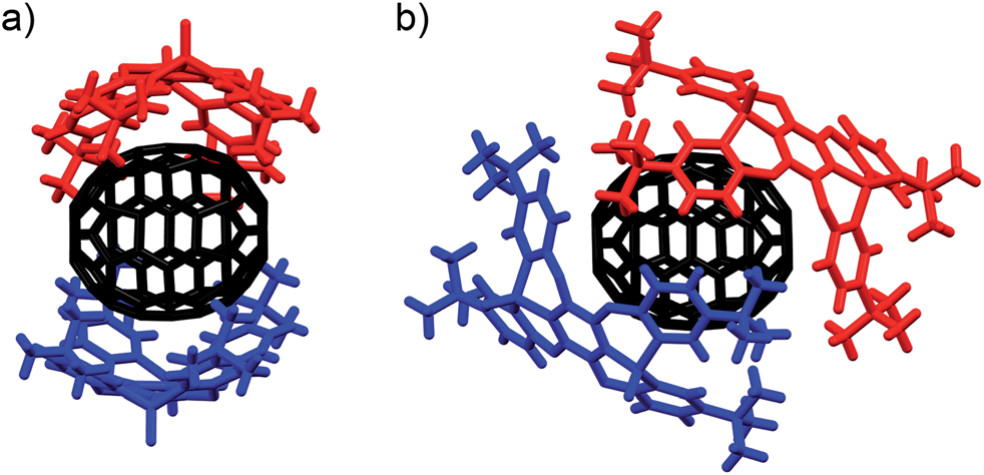
Side view of the molecular structures of the (**P2**)_2_ ⊃ C_70_ forms (a) **IV** (faulting-like) and (b) **V** (misaligned).

**Fig. 5:**
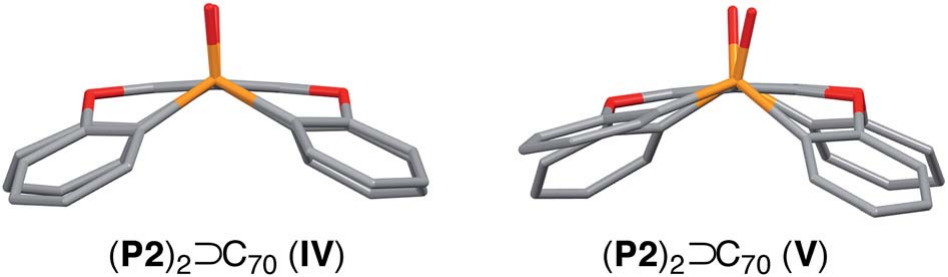
Comparison of the **P2** host moiety in **IV–V**.

Using C_70_ as a guest molecule, two different 2 : 1 host–guest complexes (**P2**)_2_ ⊃ C_70_ (**IV**, **V**) were obtained by crystallization from different solvents (Fig. 4). Similar to (**P2**)_2_ ⊃ C_60_, the two host molecules encapsulated C_70_ in a sandwich fashion. The *P*2_1_2_1_2 space group of **IV**, prepared from CHCl_3_/toluene, is non-centrosymmetric with a Flack χ value of –0.03(3). In **IV**, the P1–P2 axes of the two host molecules are almost perfectly aligned vertically with respect to the long axis of C_70_ (Fig. 4a), but the centroids of the **A** rings of the two **P2** molecules are slightly misaligned, resulting in a faulting-like chiral architecture of **IV**. Conversely, crystal **V**, obtained from CHCl_3_/CS_2_, crystallises in the centrosymmetric space group *C*2/*c*. In **V**, the two host molecules encapsulated C_70_ with their P1–P2 axes offset by 37.1° with respect to the long axis of C_70_ (Fig. 4b), and the two **P2** molecules are also misaligned. In **IV**, the torsion angle range between the central (**A**) and peripheral benzene rings (**B–E**) of **P2** (29.1–33.8°) was significantly narrower than that in **V** (13.2–37.3°) (Table 2). Similarly to the (**P2**)_2_ ⊃ C_60_ host–guest complexes, the torsion angles are flexible and are thus able to facilitate different host–guest architectures (Fig. 5). To the best of our knowledge, no reports exist on concave hosts encapsulating fullerene guests in such a variable fashion.

**Table 2:** Structural parameters of the concave moiety in **IV–V**

	(**P2**)_2_ ⊃ C_70_ (**IV**)	(**P2**)_2_ ⊃ C_70_ (**V**)
**Bowl depth**		
*d* (Å)	2.05	1.83
*D* (Å)	4.16	3.47

**Torsion angles**		
**A–B** (°)	31.9	13.2
**A–C** (°)	29.1	30.3
**A–E** (°)	29.6	37.3
**A–F** (°)	33.8	30.5

**Fig. 6:**
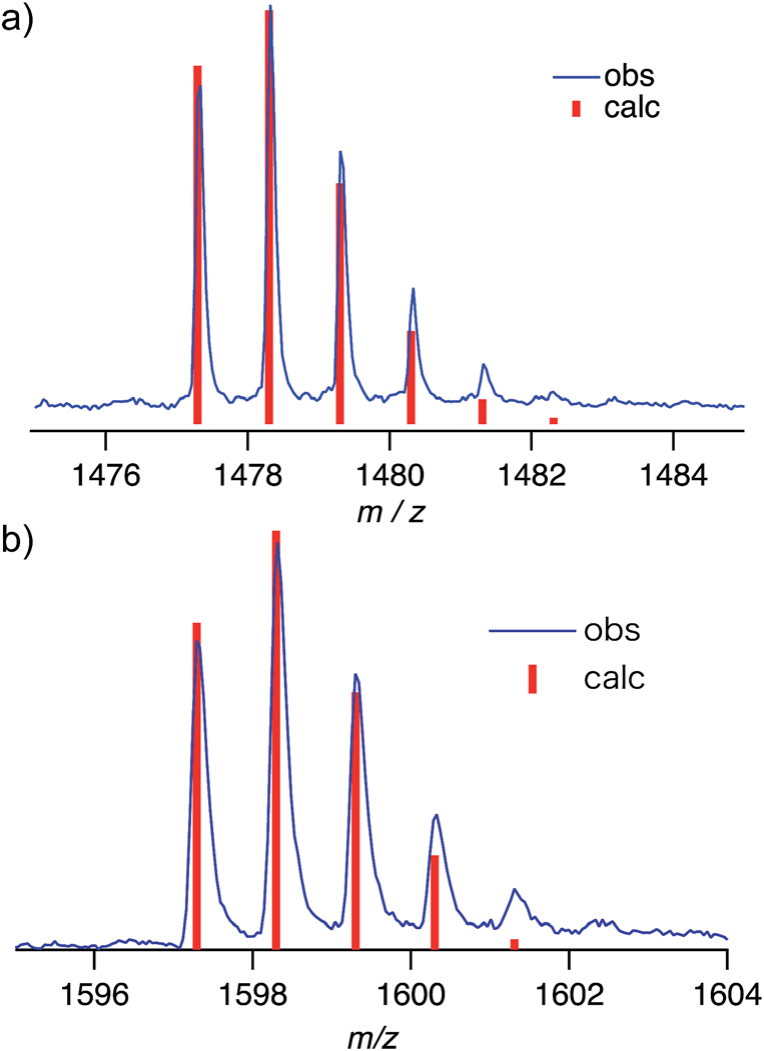
MALDI TOF MS of (a) [**P2** ⊃ C_60_–H]^–^ and (b) [**P2** ⊃ C_70_–H]^–^.

**Fig. 7:**
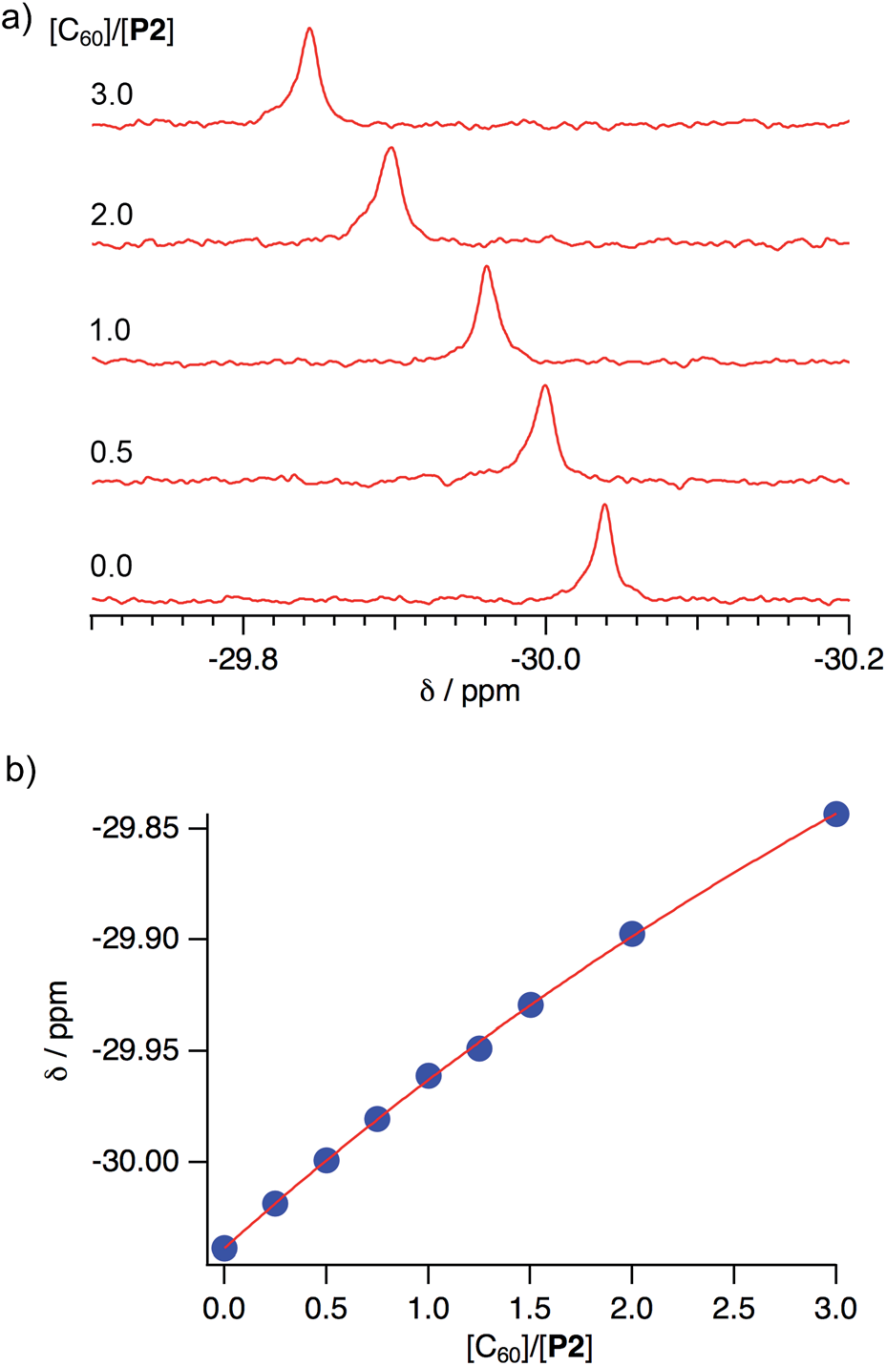
(a) Spectral changes in the ^31^P NMR spectra (243 MHz) of **P2** in CDCl_3_/CS_2_ (1 : 3 v/v) upon addition of C_60_, and (b) 1 : 1 binding isotherm.

Negative-mode MALDI-TOF mass spectra of (**P2**)_2_ ⊃ C_60_ and (**P2**)_2_ ⊃ C_70_ revealed prominent peaks at *m*/*z* = 1478.3 and 1598.3, which were assigned to the corresponding 1 : 1 complexes (Fig. 6). This result suggested that the concave–convex interactions between hosts and guests should be strong enough to preserve the host–guest complex at least partially even during ionization. However, no peaks corresponding to the 2 : 1 complexes (**P2**)_2_ ⊃ C_60_ and (**P2**)_2_ ⊃ C_70_ were observed, even though this stoichiometry was found in the crystal structure. This result indicated that the interactions with the second host molecules in the 2 : 1 complexes might be too weak in order to be observed. Therefore, NMR titration experiments were carried out in order to evaluate the concave–convex interactions in the binary organic solvent mixture CDCl_3_/CS_2_ (1 : 3 v/v). In the ^31^P NMR spectra, the signal for **P2** experienced a downfield shift (Δ*δ*_P_: 0.20 ppm) upon addition of three equivalents of C_60_ (Fig. 7a).

In the ^1^H NMR spectra, signals for the protons attached to the peripheral benzene rings were shifted slightly upfield (Δ*δ*_H_ for H_a_: –0.0049; H_b_: –0.0038; H_c_: 0.0028 ppm; see Scheme 1 and Fig. S21†). This result clearly demonstrated the interactions of **P2** with C_60_ in this solvent mixture, while the interaction between the *anti*-isomer **P2′** or previously reported **P1** and C_60_ was observed to be negligible. A Job plot analysis confirmed the formation of 1 : 1 complexes between **P2** and C_60_ (Fig. S19†), and the absence of any indications for the formation of a 2 : 1 complex is consistent with the results obtained from mass spectrometry measurements. Non-linear least-squares analysis for the change of the chemical shift afforded association constants (*K*_a_) between **P2** and C_60_ of 210 ± 20 M^–1^ (Fig. 7b),^19^ while between **P2** and C_70_ a *K*_a_ value of 200 ± 30 M^–1^ was estimated (Fig. S22†). Accordingly, **P2** exhibited an enhanced affinity for fullerenes relative to **P1**, containing one phosphorus atom, even though no selectivity for either C_60_ or C_70_ was observed. This result is consistent with the observation of multiple host–guest architectures in host–guest complexes between **P2** and fullerenes, which may reflect that **P2** is unable to selectively recognize the convex surface of specific fullerenes, due to its ability to accommodate various structures in accordance with the guest shape. A comparison of the observed *K*_a_ values with those of previously reported concave hosts, such as calixarenes and their analogues confirmed that **P2** exhibits a moderate affinity towards C_60_.^20^

**Fig. 8:**
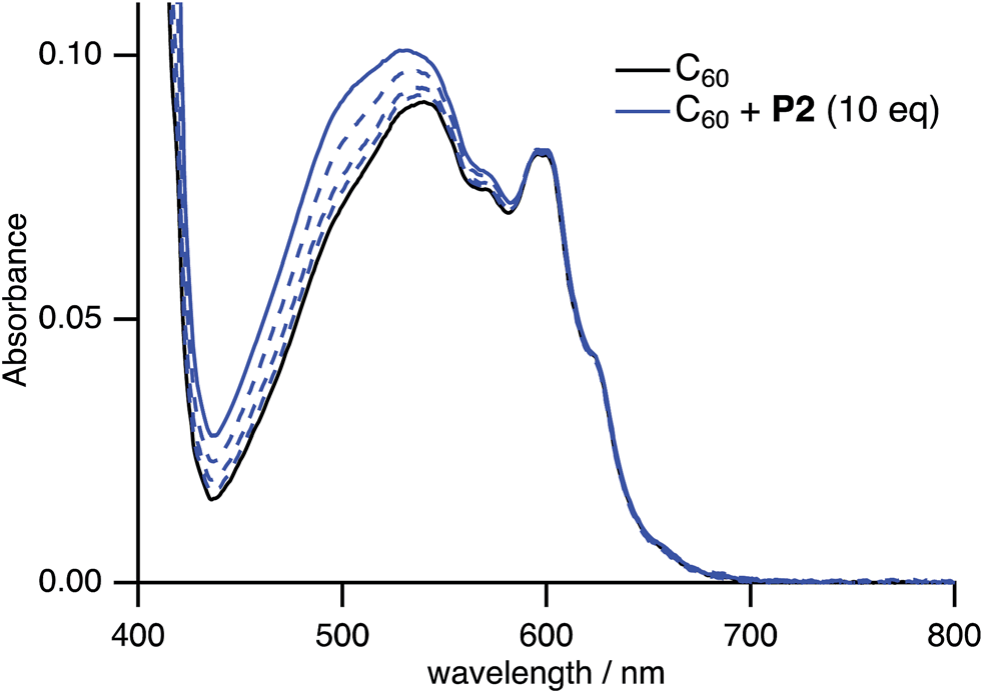
Change of the UV-vis absorption spectrum of C_60_ in CHCl_3_/toluene (1 : 4 v/v) upon addition of **P2** ([C_60_] = 0.10 mM, 0 ≤ [**P2**]/[C_60_] ≤ 10).

Spectral titration experiments based on the UV-vis absorption also confirmed the formation of host–guest complexes in solution. Upon addition of **P2** to a CHCl_3_/toluene solution of C_60_, the weak absorption at 450–600 nm, which is associated with the forbidden excitation of C_60_, gradually increased (Fig. 8), while **P2** is transparent in this region (Fig. S18†). In contrast, addition of **P2′** to a CHCl_3_/toluene solution of C_60_ did not change the UV-vis absorption spectrum (Fig. S25†). This result suggested that the stronger host–guest interaction between **P2** and C_60_ relative to **P2′** and C_60_ is responsible for the spectral change.

## Conclusions

In this study, we have disclosed the synthesis of the extended concave host **P2**, containing two phosphorus atoms. Moreover, we have demonstrated that **P2** may serve as a suitable host for fullerenes. In co-crystals with C_60_ and C_70_, two molecules of **P2** bind to the convex surface of the fullerenes in a sandwich fashion. Interestingly, the orientation of the two **P2** molecules with respect to each other is flexible, resulting in the formation of a variety of cavity shapes. MALDI-TOF mass, NMR, and UV-vis absorption spectra supported the formation of 1 : 1 host–guest complexes between **P2** and the fullerenes in solution. Relative to **P1** with one phosphorus atom, the affinity of **P2**, containing two phosphorus atoms, towards fullerenes was significantly enhanced. Accordingly, the expansion of the concave surface by fusing two phosphorus-containing concave units should result in an effective recognition of fullerenes. Considering that two molecules of **P2** are necessary for the encapsulation of fullerene, fusion of two **P2** molecules should enhance the affinity even further. Control over the cavity shape should be possible *via* a judicious choice of the connecting moiety between these two **P2** units. Studies in this direction are currently in progress in our laboratory.

## Supplementary Material

Supplementary information

Crystal structure data
