# Supplementary material for: Twofold fused concave hosts containing two phosphorus atoms: modules for the sandwich-type encapsulation of fullerenes in variable cavities
Source: Chem Sci. 2015 Jul 24;6(11):6373–8. doi: 10.1039/c5sc02224j (PMC6054080; doi:10.1039/c5sc02224j)
Supplement: Supplementary file 1 [file SC-006-C5SC02224J-s001.pdf]

Electronic Supplementary Material (ESI) for Chemical Science.
This journal is © The Royal Society of Chemistry 2015

Supporting Information

Twofold Fused Concave Hosts Containing Two Phosphorus Atoms: Modules for the Sandwich-type Encapsulation of Fullerenes in Variable Cavities

Masaki Yamamura,[a]* Daigo Hongo,[a] Tatsuya Nabeshima[a]*

[a] *Graduate School of Pure and Applied Sciences, Tsukuba Research Center for Interdisciplinary Materials Science (TIMS), University of Tsukuba*
1-1-1, Tennodai, Tsukuba, Ibaraki 305-8571, Japan

E-mail: myama@chem.tsukuba.ac.jp

Table of contents

(1) General methods S2

(2) Synthetic procedure S2

(3) NMR spectra S4

(4) MALDI-TOF mass spectra S12

(5) UV-vis spectra S13

(6) NMR spectral titration S14

(7) UV-vis spectral titration S19

(8) X-ray Crystallographic Analysis S20

(9) References S27

(1) General methods

All chemicals were reagent grade, and used without further purification. All reactions were performed under a nitrogen atmosphere. Chromatography was performed using SiO_2-60N (0.063–0.212 mm; Kanto). Melting points were determined using a Yanaco and are uncorrected. The ^{1}H, ^{13}C, ^{19}F, and ^{31}P NMR spectra were recorded by Bruker AVANCE400 (400 MHz) or AVANCE600 (600 MHz) spectrometer at 298 K. Deuterated solvents were purchased from Cambridge Isotope Laboratories or Aldrich and used as received. Tetramethylsilane was used as the internal standard (0 ppm) for ^{1}H and ^{13}C NMR. Hexafluorobenzene was used as the external standard (–162 ppm) for ^{19}F NMR. Phosphorous acid was used as the external standard (0 ppm) for ^{31}P NMR. MALDI-TOF mass spectra were recorded by an AB Sciex TOF/TOF5800. UV-vis absorption spectra were recorded by JASCO Ubest V-670 at 298 K.

(2) Synthetic procedure

Synthesis of 1

Compound **1** was prepared according to the synthetic procedure for similar derivatives.[S1] To sodium suspension (0.56 g, 24 mmol) in THF (60 mL) was added ethyl phosphite (2.0 mL, 15 mmol) and then the mixture was stirred at ambient temperature for 38 hours. This was added at 0 °C to an ether (30 mL) solution of 2-lithio-4-*tert*-butylanisole, which was prepared from 4-*tert*-butylanisole (5.5 g, 33 mmol), TMEDA (0.50 mL, 0.39 g, 3.4 mmol) and *n*-butyllithium/hexane (2.65 M, 13.5 mL, 35.8 mmol). The reaction mixture was stirred at ambient temperature for further 2 hours. This was quenched with 1 M HCl, and then most of solvent was evaporated. The residue was extracted with dichloromethane forth, and the combined organic phase was dried over $MgSO_4$. Evaporation of the solvent gave the crude material. The crude material was washed with ether to give colorless powder of **1**. The filtrate was evaporated and purified by silica-gel column chromatography (eluent: $CHCl_3$) to give colorless powder of **1** (total yield: 2.6 g, 6.9 mmol, 45 %).

1: colorless powder, mp. 158 °C; ^{1}H NMR (400 MHz, $CDCl_3$) δ□1.30 (s, 18H), 3.70 (s, 6H), 6.80 (dd, ^{3}J = 8.5 Hz, J = 6.0 Hz, 2H), 7.48 (dd, ^{3}J = 8.5 Hz, J = 2.4 Hz, 2H) , 7.73 (dd, J = 2.4 Hz, $^{3}J_{HP}$ = 16.3 Hz, 2H) , 8.21 (d, $^{1}J_{HP}$ = 509.4 Hz, 1H); ^{13}C NMR (100MHz, $CDCl_3$) δ 31.41 (CH_3, s), 34.28 (C, s), 55.60 (CH_3, s), 110.30 (*C*H, d, J = 6.6 Hz), 119.44 (*C*, d, J = 104.3 Hz), 130.20 (*C*H, d, J = 8.9 Hz), 130.40 (*C*H, d, J = 2.0 Hz), 143.34 (*C*, d, J = 11.7 Hz), 158.63 (*C*, d, J = 3.6 Hz); ^{31}P NMR (162 MHz, $CDCl_3$) δ 11.97 (s); MALDI-TOF MS *m/z* 375.25 $[M+H]^+$; Anal. Calcd. for $C_{22}H_{31}O_3P$: C 70.57, H 8.34; found: C 70.12, H 8.47.

Synthesis of 2

To a THF (50 mL) solution of **1** (1.50 g, 4.01 mmol) was added *n*-butyllithium/hexane (2.65 M, 1.5 mL, 4.0 mmol) at 0 °C and then the mixture was stirred at 0 °C for 2 hours. This was added to a THF (10 mL) solution of hexafluorobenzene (0.23 mL, 0.37 g, 2.0 mmol) at 0 °C and then the mixture was stirred at ambient temperature for 16 hours. This was quenched with water, and then most of THF was evaporated. The residue was extracted with dichloromethane forth, and the combined organic phase was dried over $MgSO_4$. Evaporation of the solvent gave the crude material. The crude material was precipitated from ether/hexane to give colorless powder of **2** (0.461 g, 0.515 mmol, 26 %).

2: colorless powder, mp. >280 °C; ^{1}H NMR (600 MHz, $CDCl_3$) δ 1.23 (s, 36H), 3.63 (s, 12H), 6.84 (dd, ^3J = 8.6 Hz, J = 6.2 Hz, 4H), 7.54 (dd, ^3J = 8.6 Hz, J = 2.4 Hz, 4H), 7.59 (dd, J = 2.4 Hz, $^3J_{HP}$ = 16.2 Hz, 4H); ^{13}C NMR (150 MHz, $CDCl_3$) δ 31.32 (CH$_3$, s), 34.30 (C, s), 55.52 (CH$_3$, s), 110.37 (CH, d, J = 7.2 Hz), 118.31 (C, d, J = 92.2 Hz), 118.78 (C, d, J = 115.1 Hz), 130.69 (CH, d, J = 9.5 Hz), 131.09 (CH, s), 143.41 (C, d, J = 12.3 Hz), 146.58 (C, dd, J = 253.2 Hz, J = 13.7 Hz), 159.18 (C, d, J = 2.2 Hz); ^{31}P NMR (243 MHz, $CDCl_3$) δ 15.64 (s); ^{19}F NMR (565 MHz, $CDCl_3$) δ –133.7 (s); MALDI-TOF MS *m/z* 895.43 [M+H]$^+$; Anal. Calcd. for $C_{50}H_{60}F_4O_6P_2$: C 67.10, H 6.76; found: C 66.93, H 6.80.

Synthesis of 3

To a dichloromethane (20 mL) solution of **2** (359 mg, 401 μmol) was added boron tribromide (0.60 mL, 6.3 mmol) at ambient temperature and then the mixture was stirred for 8 hours. This was poured into ice/water. The mixture was extracted with dichloromethane, and the combined organic phase was dried over $MgSO_4$. Evaporation of the solvent gave the crude material. The crude material was washed with ether to give colorless powder of **3** (304 mg, 363 μmol, 91%).

3: colorless powder, mp. >280 °C; ^{1}H NMR (400 MHz, acetone-d_6) δ 1.24 (s, 36H), 6.95 (dd, ^3J = 8.7 Hz, J = 6.3 Hz, 4H), 7.60 (dd, ^3J = 8.7 Hz, J = 2.0 Hz, 4H), 7.77 (dd, J = 2.0 Hz, $^3J_{HP}$ = 16.3 Hz, 4H), 10.32 (s, 2H); ^{13}C NMR (100 MHz, acetone-d_6) δ 30.70 (CH$_3$, s), 33.93 (C, s), 113.71 (C, d, J = 113.0 Hz), 116.76 (CH, d, J = 8.8 Hz), 116.85 (CH, d, J = 8.7 Hz), 127.93 (CH, d, J = 10.6 Hz), 132.45 (CH, d, J = 2.2 Hz), 142.38 (C, d, J = 12.4 Hz), 146.63 (C, d, J = 237.2 Hz), 158.68 (C, d, J = 4.2 Hz), 158.82 (C, d, J = 3.6 Hz); ^{31}P NMR (162 MHz, acetone-d_6) δ 27.0 (s); ^{19}F NMR (376 MHz, acetone-d_6) δ −133.8 (s); MALDI-TOF MS *m/z* 839.41 [M+H]$^+$; Anal. Calcd. for $C_{46}H_{52}F_4O_6P_2 \cdot H_2O$: C 64.48, H 6.35; found: C 64.17, H 6.18.

Synthesis of P2

To a DMF (80 mL) solution of **3** (136 mg, 162 μmol) was added *t*-BuOK (90.0 mg, 0.80 mmol) at ambient temperature and then the mixture was stirred at 80 ºC for 44 hours. To the reaction mixture was added 1 M HCl at 0 ºC. The mixture was extracted with dichloromethane, and the combined organic phase was dried over $MgSO_4$. Evaporation of the solvent gave the crude material. The crude material was separated by silica-gel column chromatography to give colorless powder of **P2** (93.7 mg, 123 μmol, 76%) and **P2'** (18.2 mg, 24.0 μmol, 15%).

P2: colorless solid, mp. > 280°C; ^{1}H NMR (400 MHz, $CDCl_3$) δ 1.42 (s, 36H), 7.54 (dd, ^3J = 8.9 Hz, J = 6.4 Hz, 4H), 7.71 (dd, ^3J = 8.9 Hz, J = 2.4 Hz, 4H), 8.23 (dd, $^3J_{HP}$ = 12.7 Hz, J = 2.4 Hz, 4H); ^{13}C NMR (100 MHz, $CDCl_3$) δ 31.3 (CH_3, s), 34.8 (*C*, s), 109.1 (C, d, J = 91.3 Hz), 116.0 (C, d, J = 115.9 Hz), 119.8 (CH, d, J = 6.4 Hz), 125.6 (CH, d, J = 5.5 Hz), 131.6 (CH, s), 139.4 (C, d, J = 9.3 Hz) , 147.9 (C, d, J = 9.9 Hz), 155.5 (C, d, J = 2.2 Hz). ^{31}P NMR (162MHz, $CDCl_3$) δ –27.9 (s). MALDI-TOF MS *m/z* 758.34 $[M+H]^+$; Anal. Calcd. for $C_{46}H_{48}O_6P_2 \cdot H_2O$: C 71.12, H 6.49; found: C 71.07, H 6.29.

P2': colorless solid, mp. > 280°C; ^{1}H NMR (400MHz, $CDCl_3$) δ 1.45 (s, 36H), 7.60 (dd, ^3J = 8.9 Hz, J = 6.4 Hz, 4H), 7.75 (dd, ^3J = 8.9 Hz, J = 2.3 Hz, 4H), 8.26 (dd, $^3J_{HP}$ = 12.9 Hz, J = 2.3 Hz, 4H). ^{31}P NMR (162MHz, $CDCl_3$) δ –27.5 (s). ^{13}C NMR (100MHz, $CDCl_3$) δ 31.3 (CH_3, s), 34.8 (C, s), 108.7 (C, d, J = 90.6 Hz), 116.3 (C, d, J = 116.7 Hz), 120.0 (CH, d, J = 6.4 Hz), 125.4 (CH, d, J = 5.5 Hz), 131.7 (CH, s), 138.4 (C, d, J = 8.8 Hz) , 148.0 (C, d, J = 10.0 Hz). , 155.3 (C, d, J = 1.5 Hz). MALDI-TOF MS *m/z* 758.34 $[M+H]^+$; Anal. Calcd. for $C_{46}H_{48}O_6P_2 \cdot 1.5H_2O$: C 70.31, H 6.54; found: C 70.22, H 6.46.

(3) NMR spectra

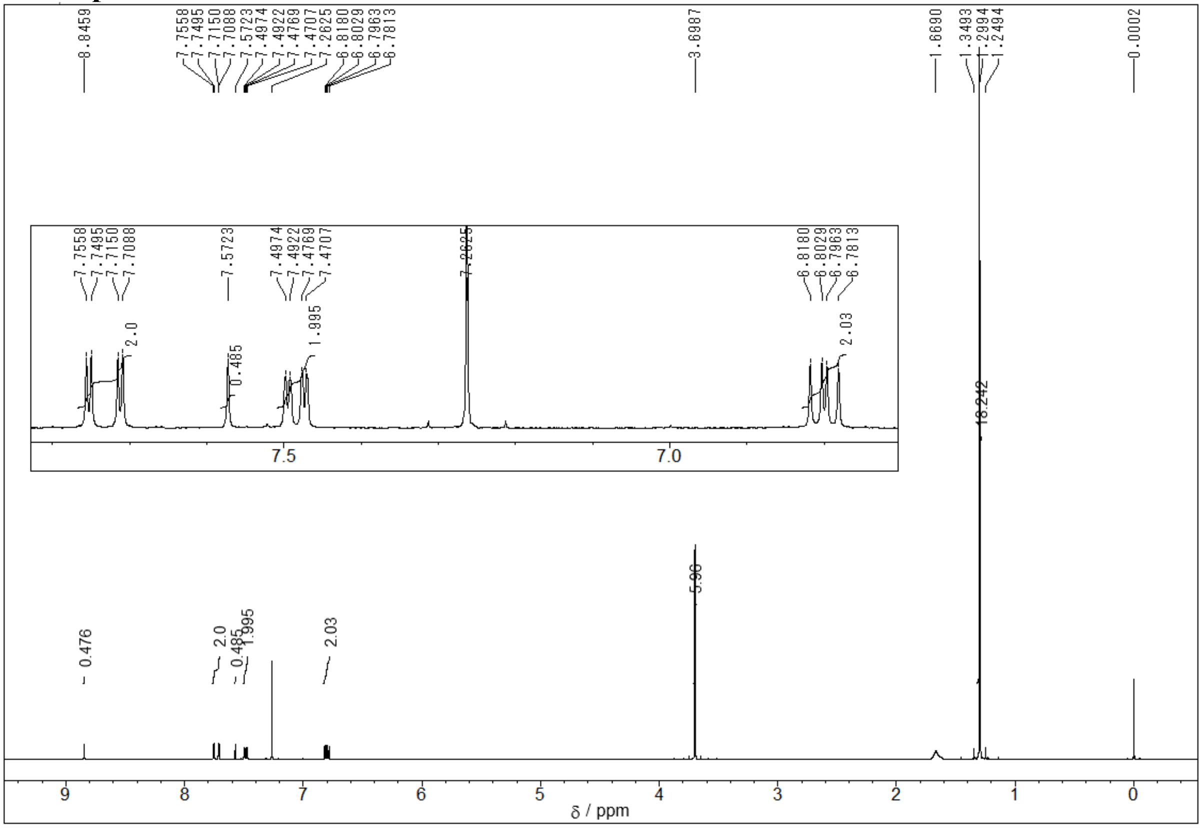

Figure S1. ^{1}H NMR spectrum of **1** (400 MHz, $CDCl_3$).

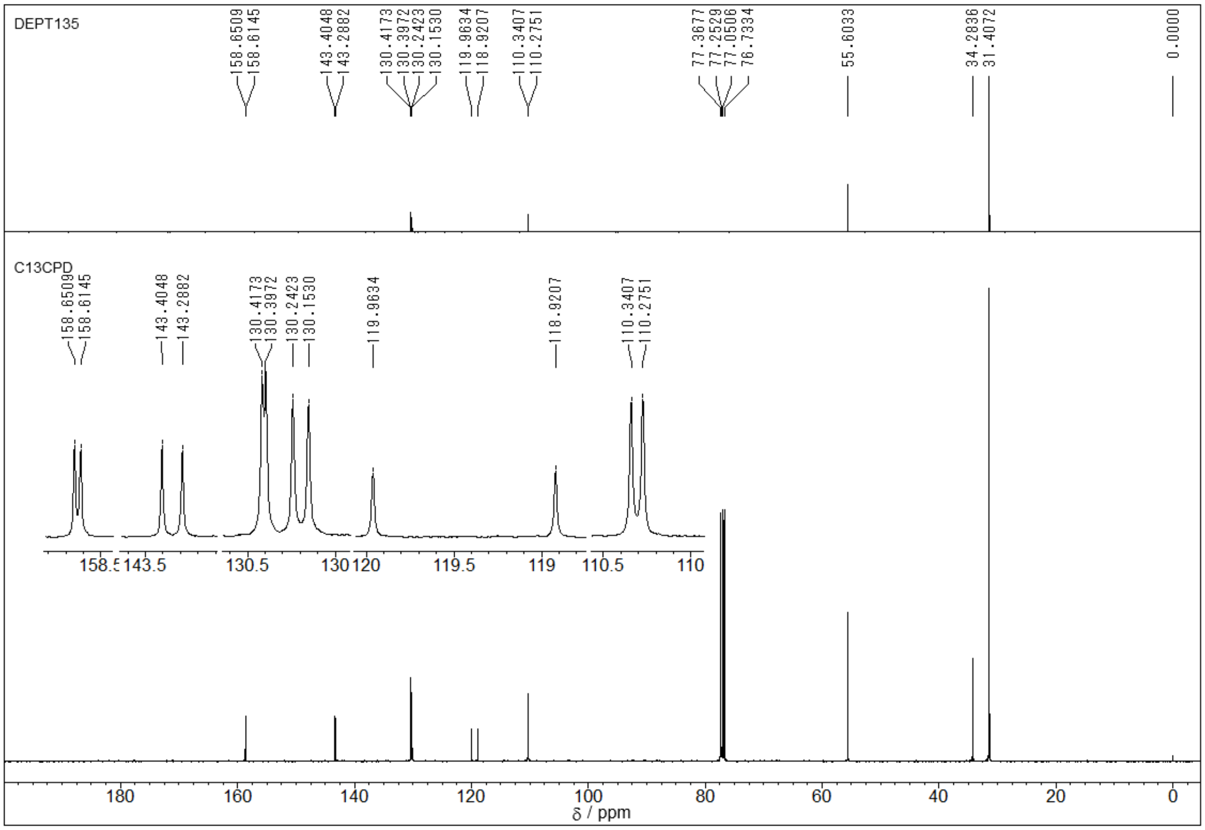

Figure S2. ^{13}C NMR spectrum of **1** (100 MHz, $CDCl_3$).

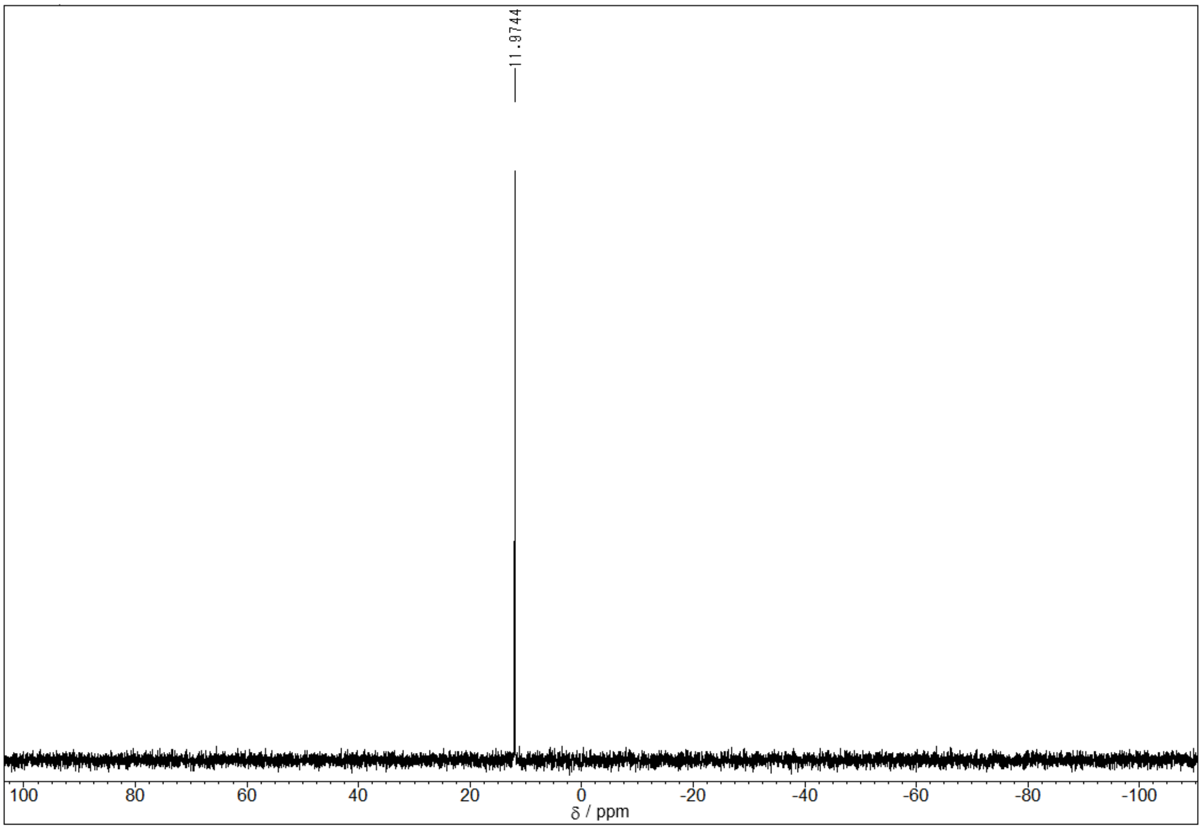

Figure S3. ^{31}P NMR spectrum of **1** (162 MHz, $CDCl_3$).

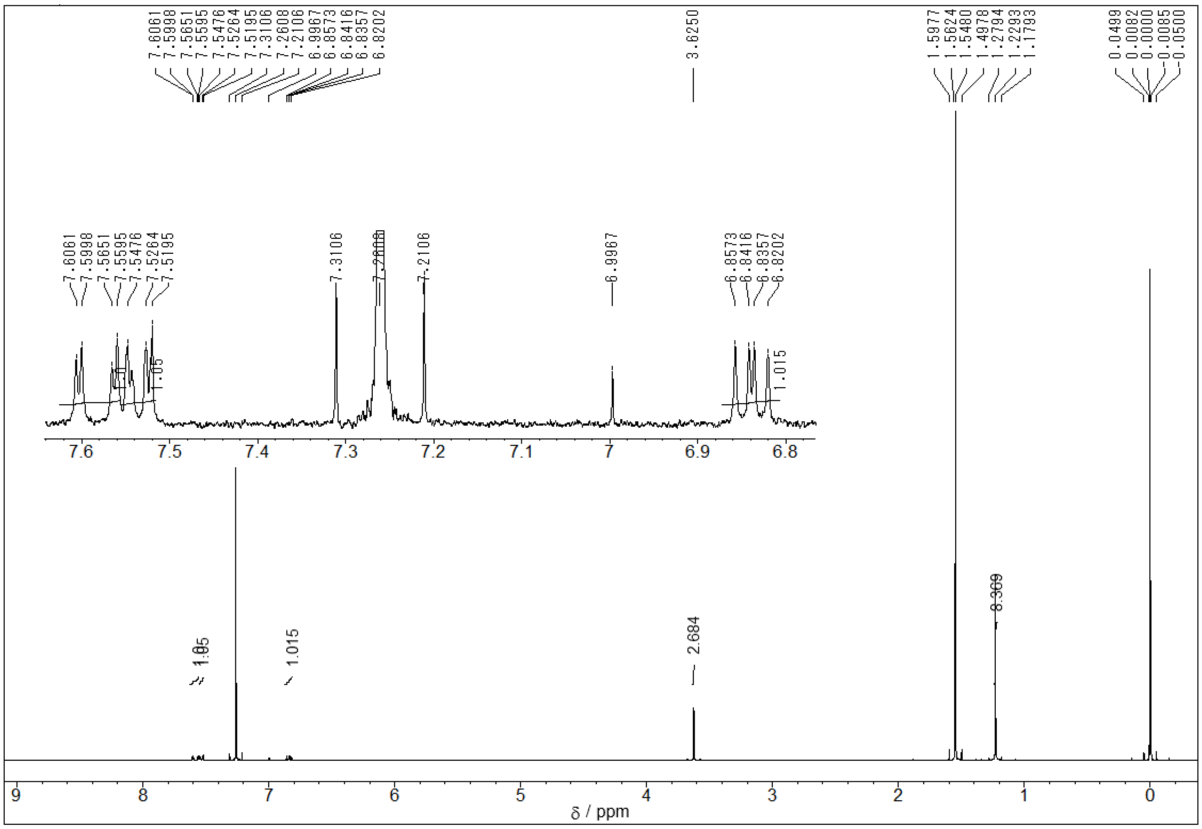

Figure S4. ^{1}H NMR spectrum of **2** (600 MHz, $CDCl_3$).

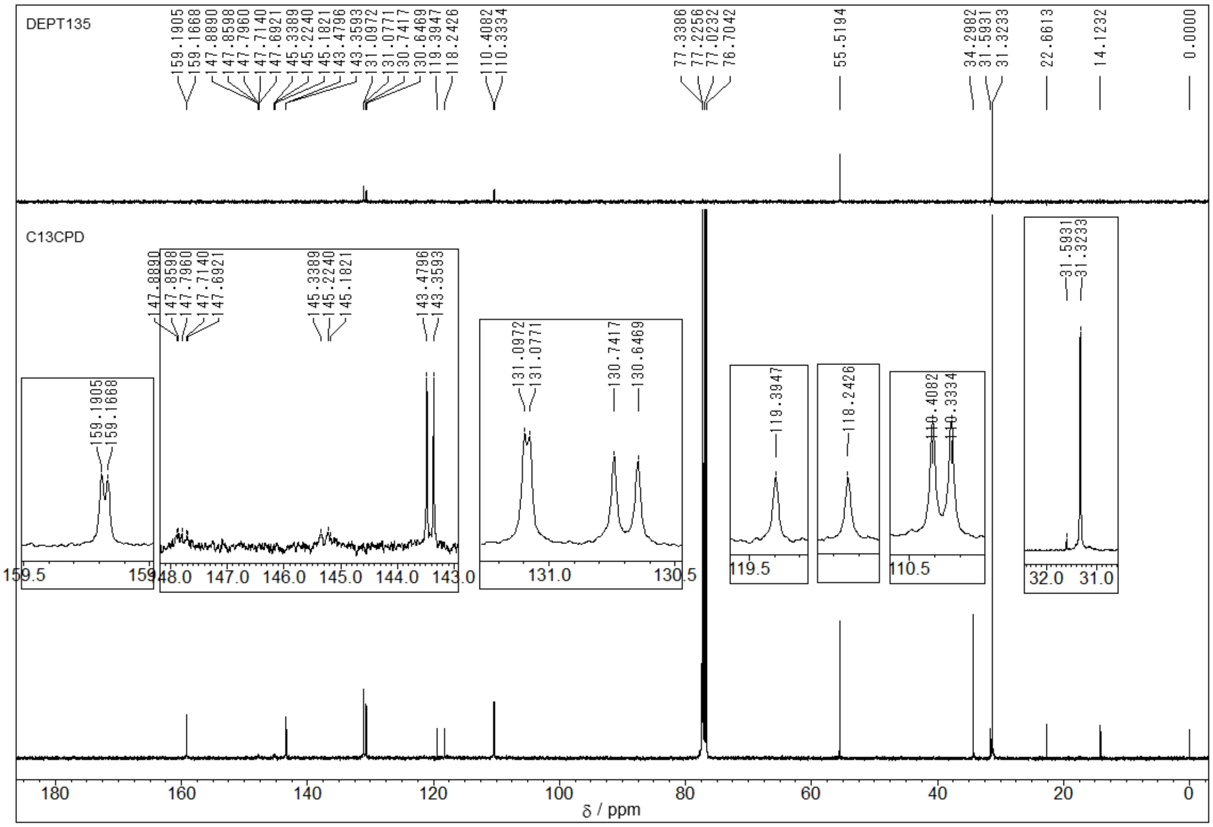

Figure S5. ^{13}C NMR spectrum of **2** (150 MHz, $CDCl_3$).

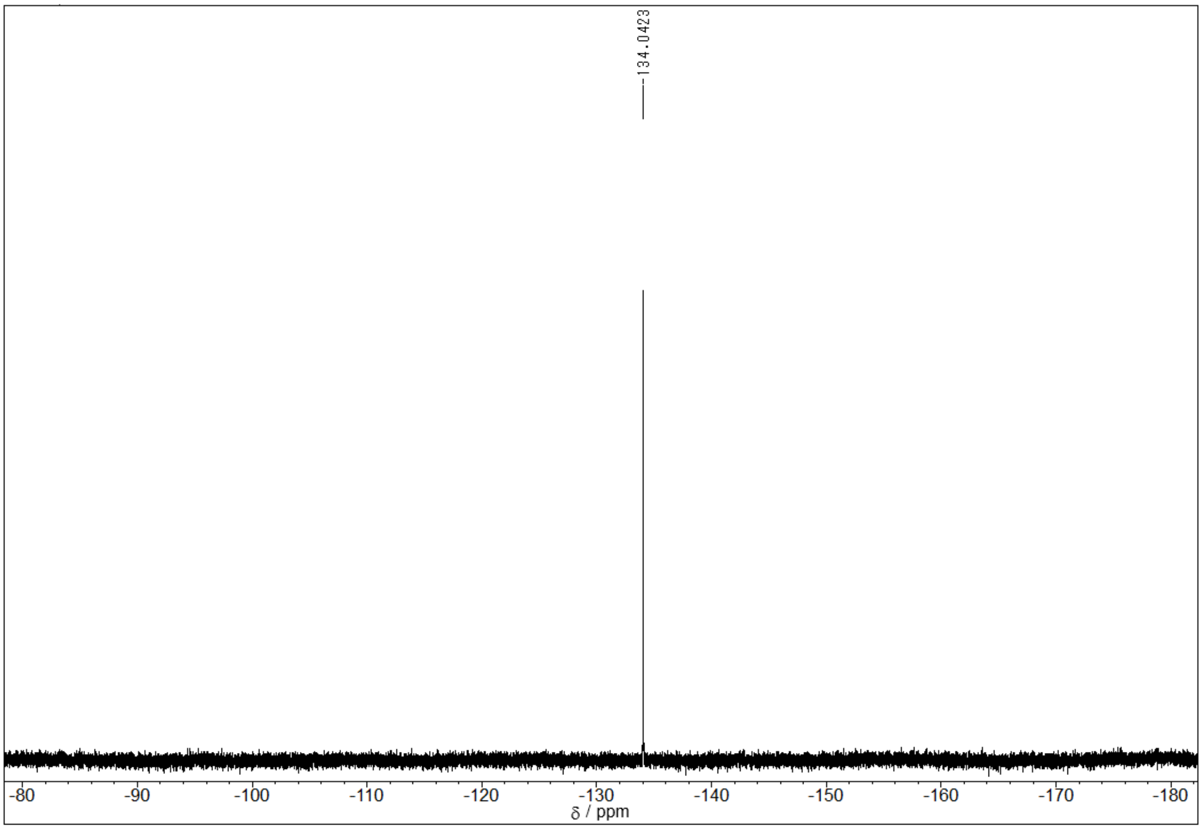

Figure S6. ^{19}F NMR spectrum of **2** (565 MHz, $CDCl_3$).

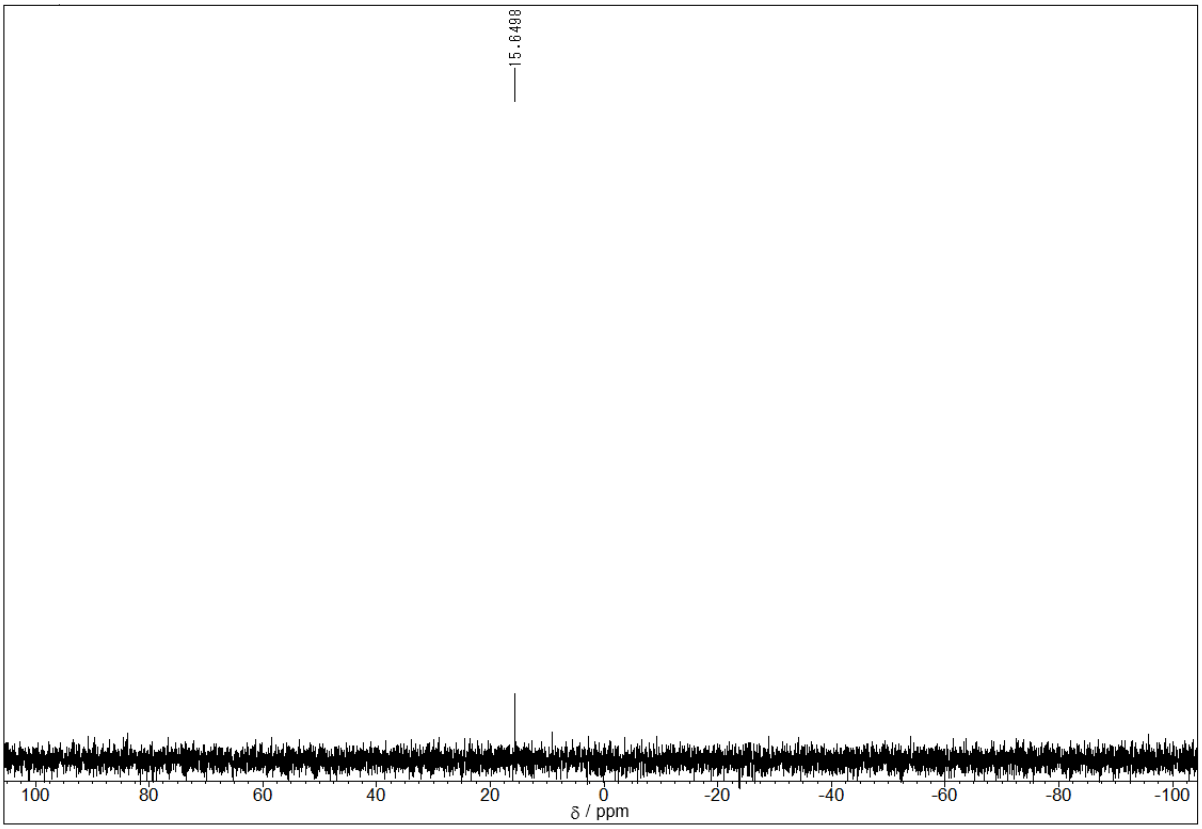

Figure S7. ^{31}P NMR spectrum of **2** (243 MHz, $CDCl_3$).

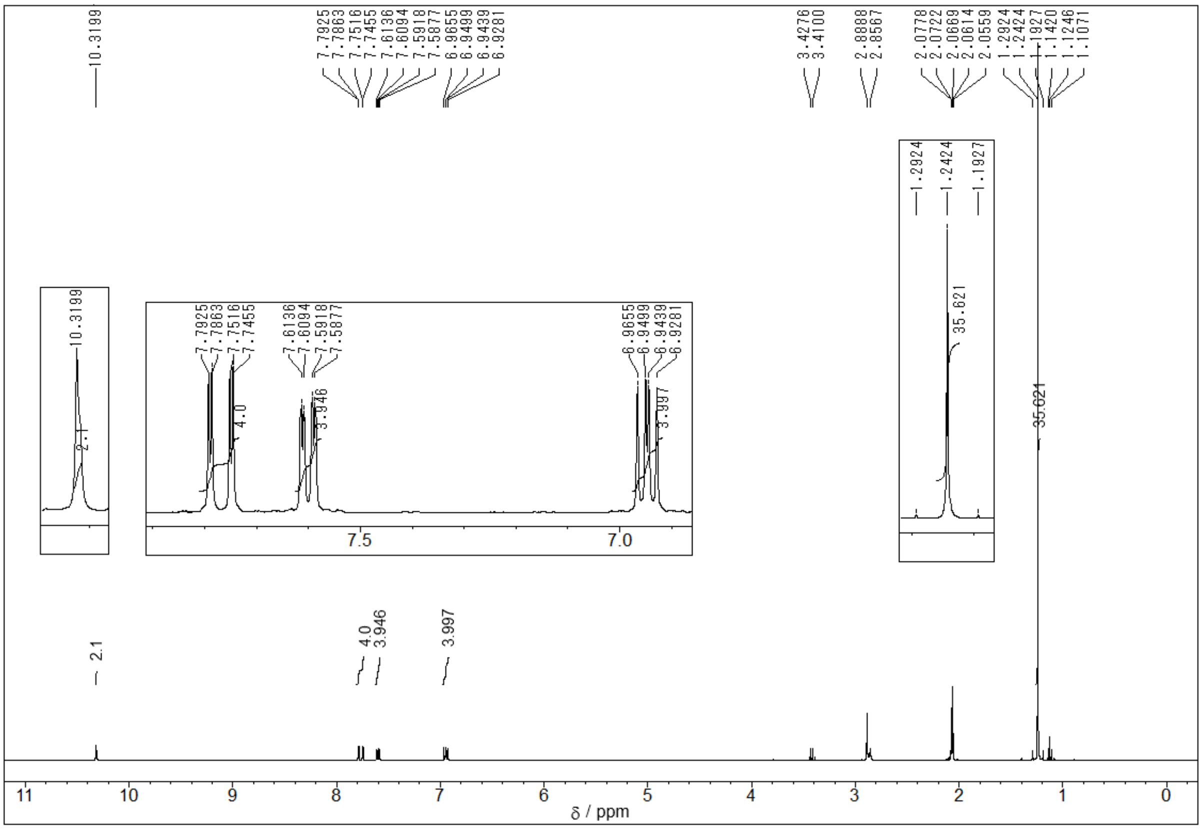

Figure S8. ^{1}H NMR spectrum of **3** (400 MHz, acetone-d_6).

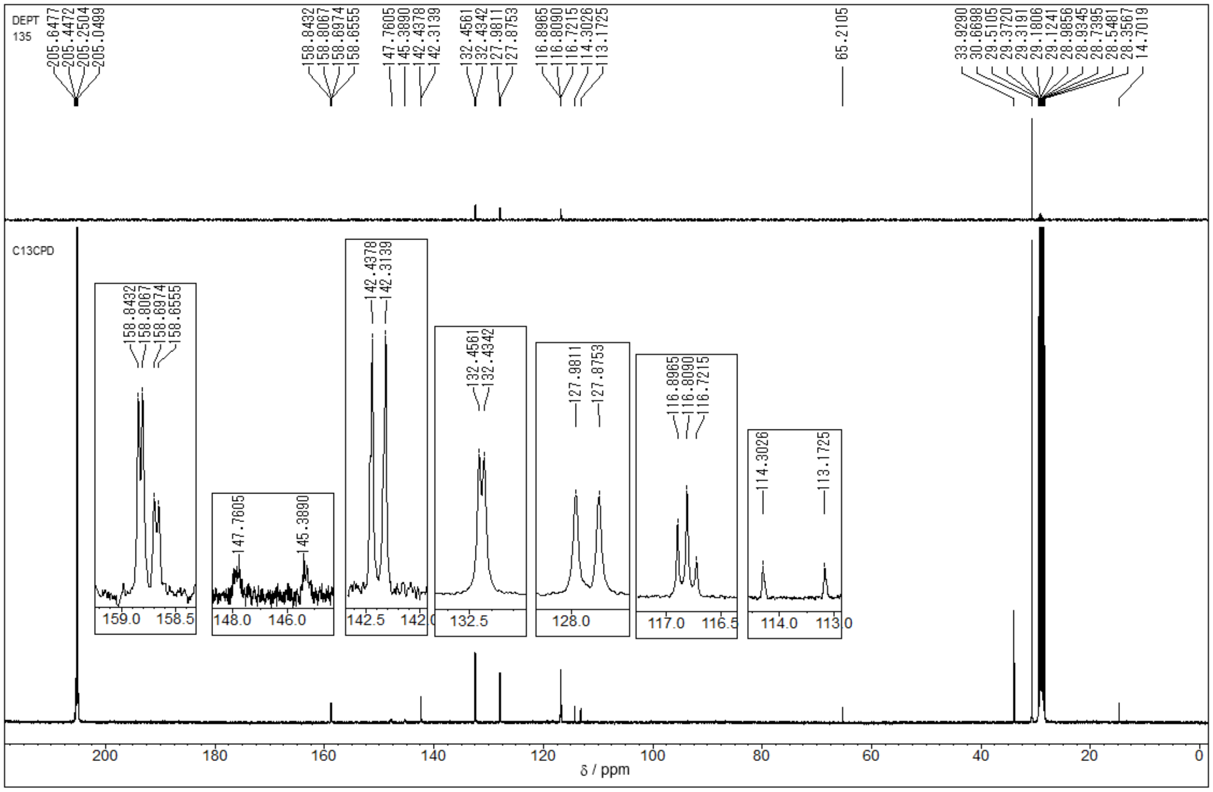

Figure S9. ^{13}C NMR spectrum of **3** (100 MHz, acetone-d_6).

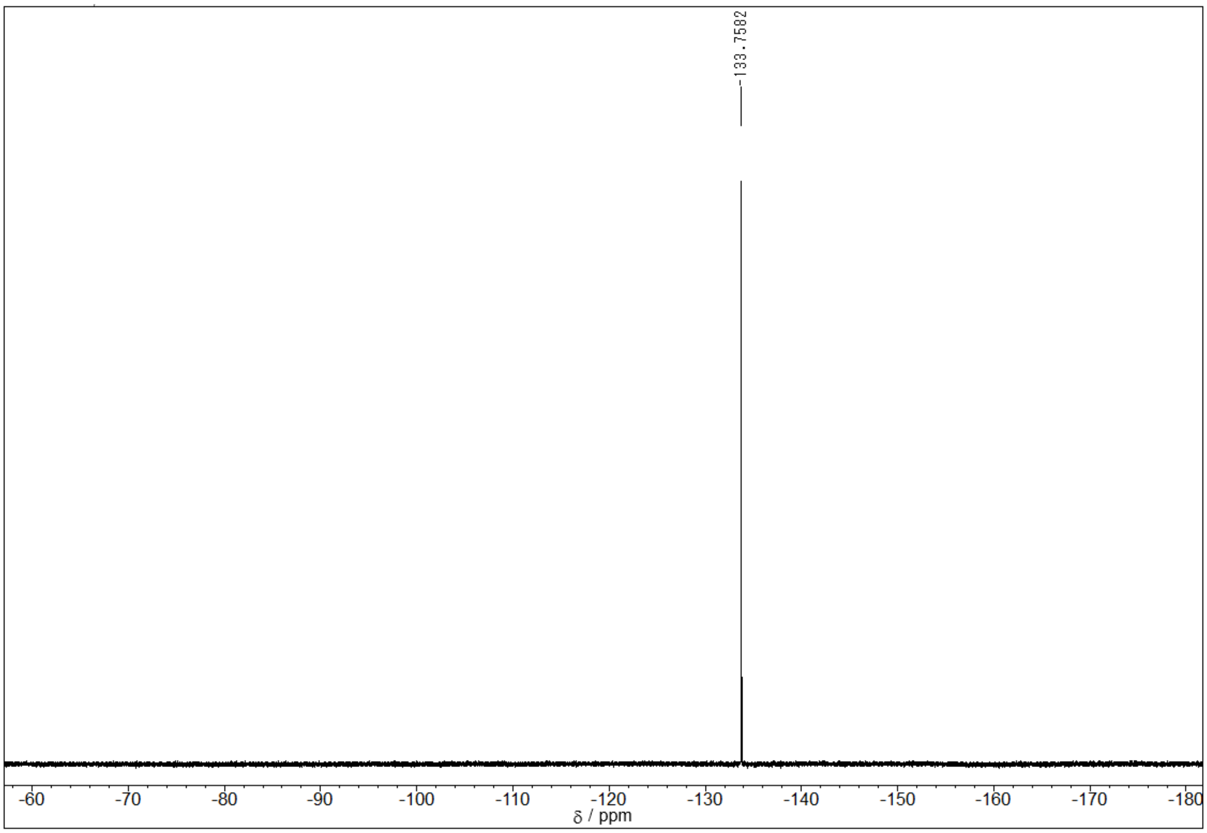

Figure S10. ^{19}F NMR spectrum of **3** (376 MHz, acetone-d_6).

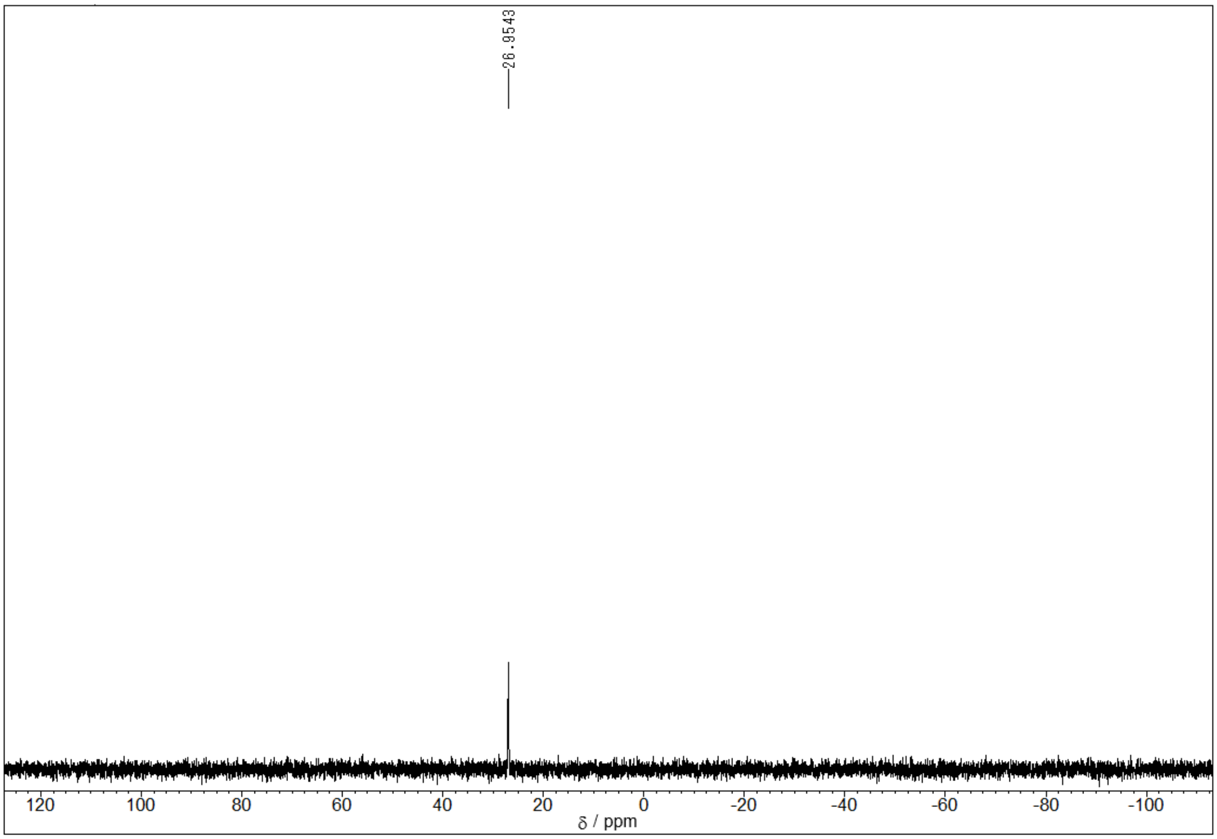

Figure S11. ^{31}P NMR spectrum of **3** (162 MHz, acetone-d_6).

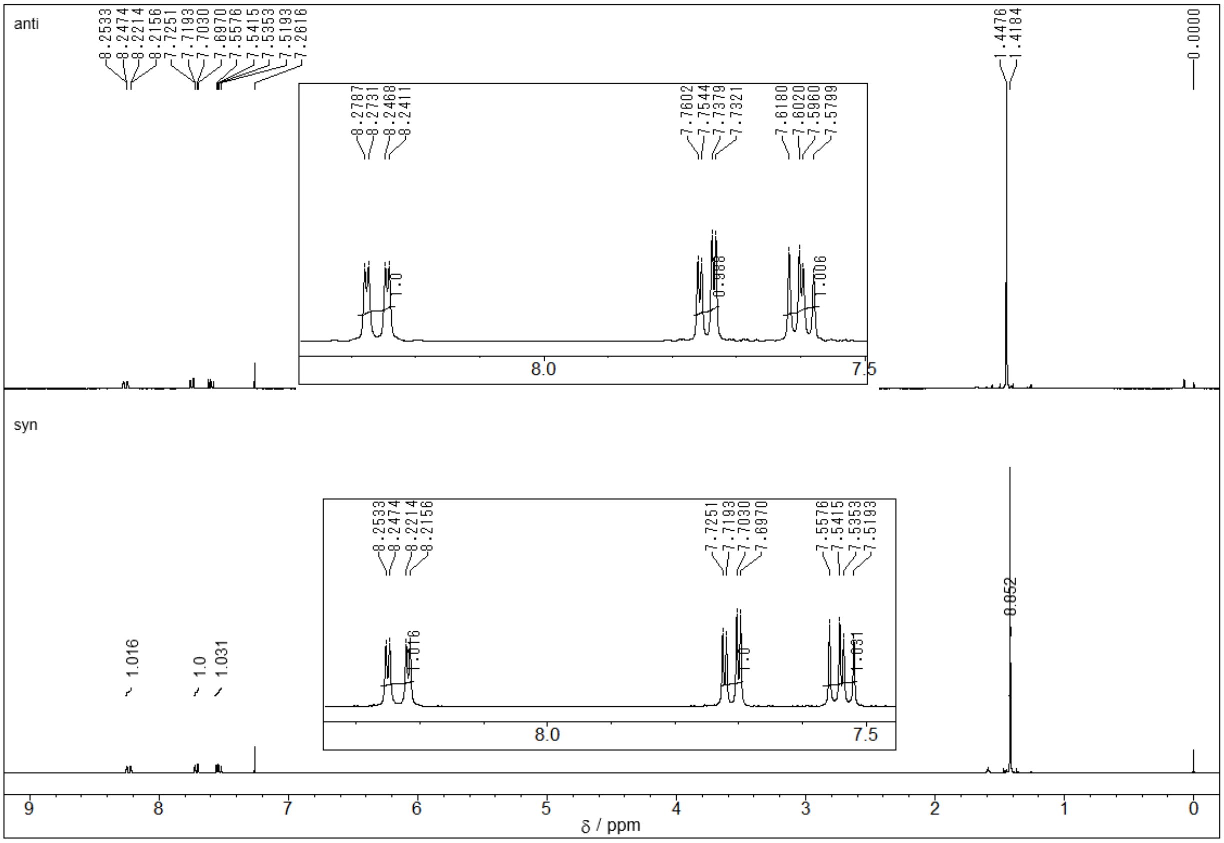

Figure S12. ^{1}H NMR spectra of **P2'** (Top) and **P2** (bottom) (400 MHz, $CDCl_3$).

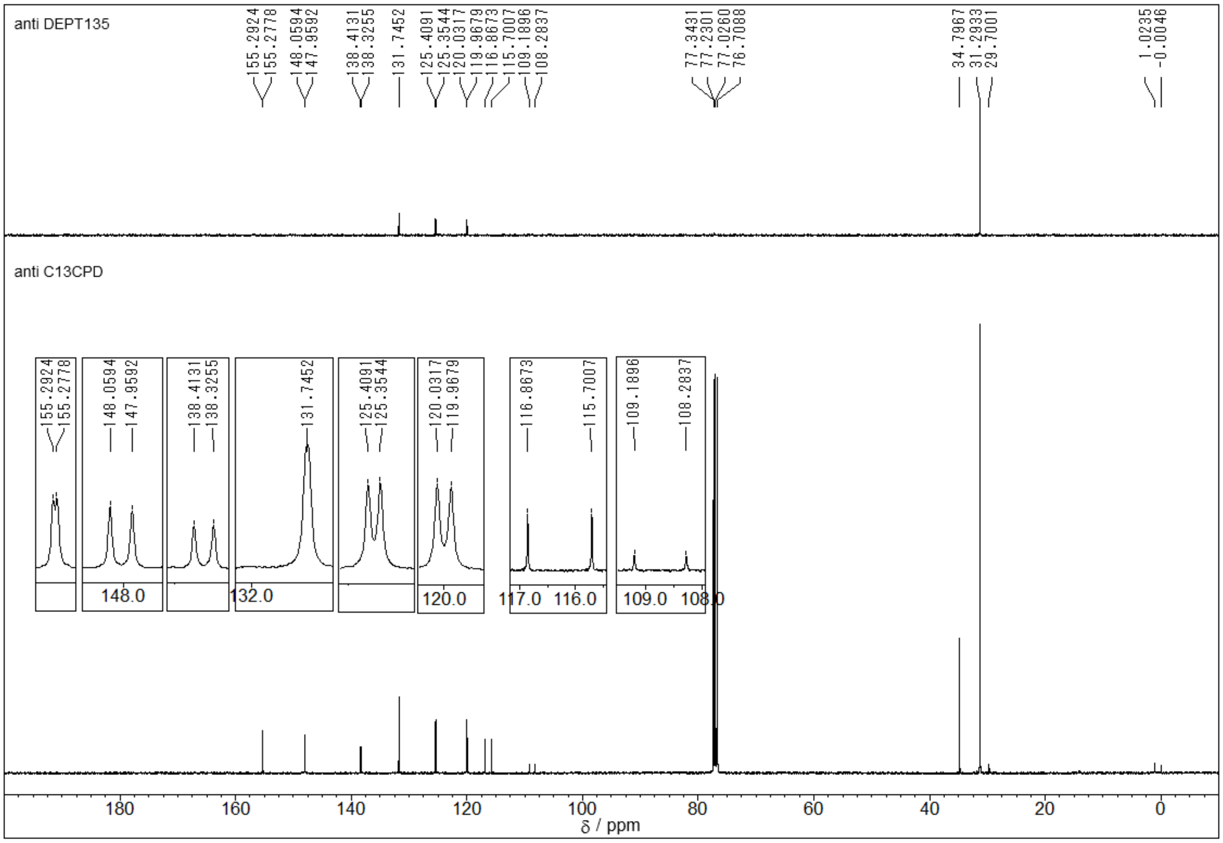

Figure S13. ^{13}C NMR spectrum of **P2'** (100 MHz, $CDCl_3$).

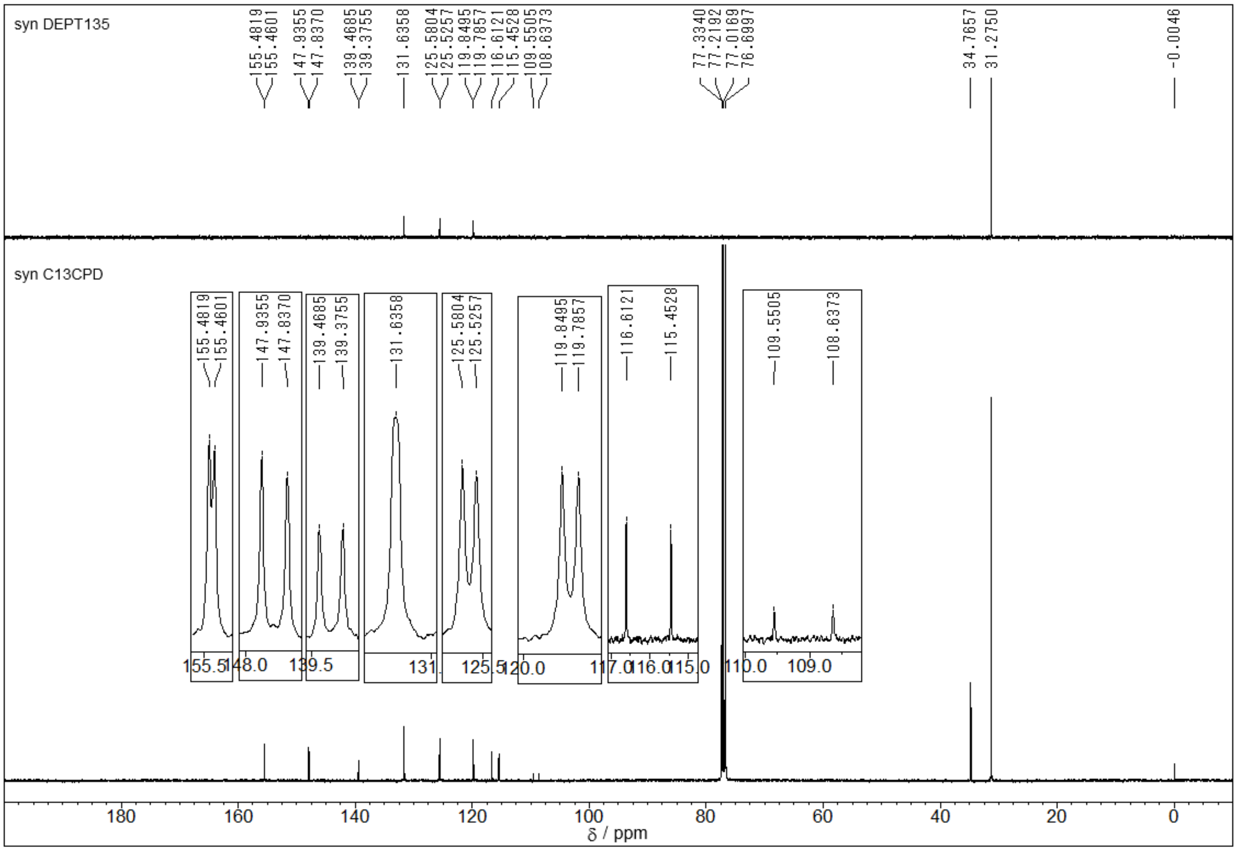

Figure S14. ^{13}C NMR spectrum of **P2** (100 MHz, $CDCl_3$).

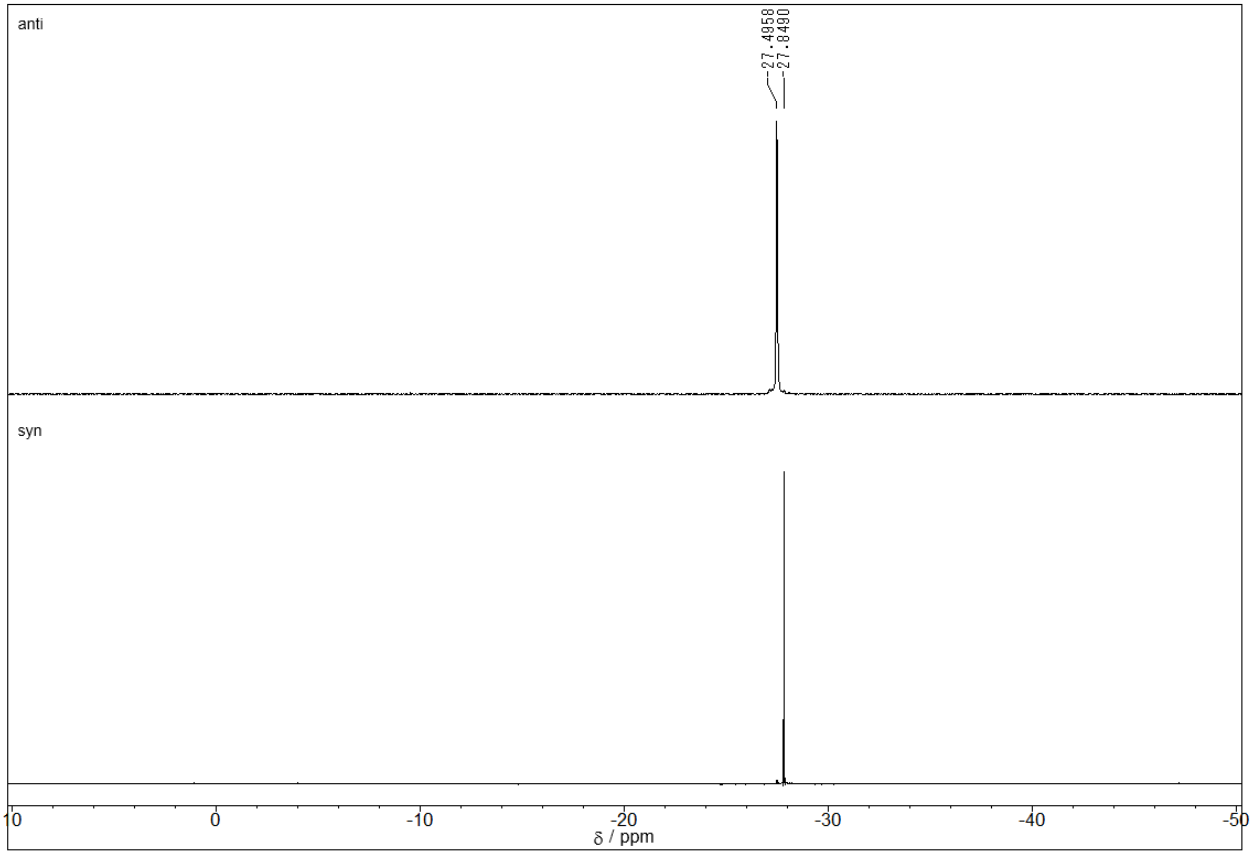

Figure S15. ^{31}P NMR spectrum of **P2’** (Top) and **P2** (bottom) (162 MHz, $CDCl_3$).

(4) MALDI TOF MS

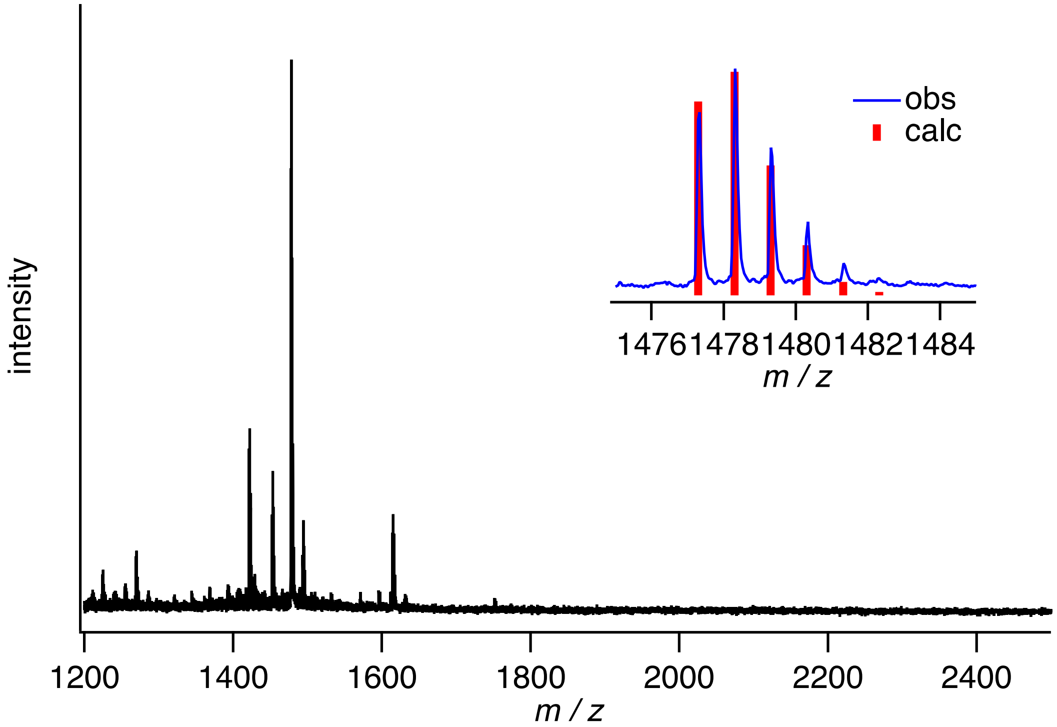

Figure S16. MALDI TOF MS of (**P2**)$_2$⊃C_{60}. Inset shows calculated isotope peaks for [**P2**⊃C_{60}–H]$^-$.

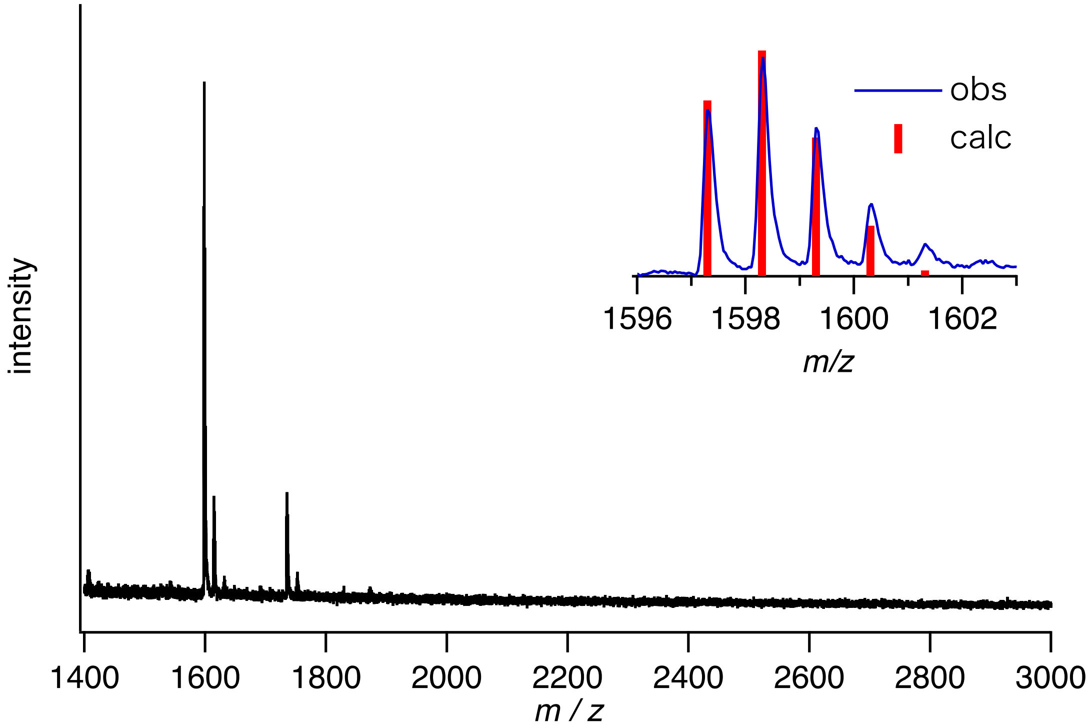

Figure S17. MALDI TOF MS of (**P2**)$_2$⊃C_{70}. Inset shows calculated isotope peaks for [**P2**⊃C_{70}–H]$^-$.

(5) UV-vis spectra

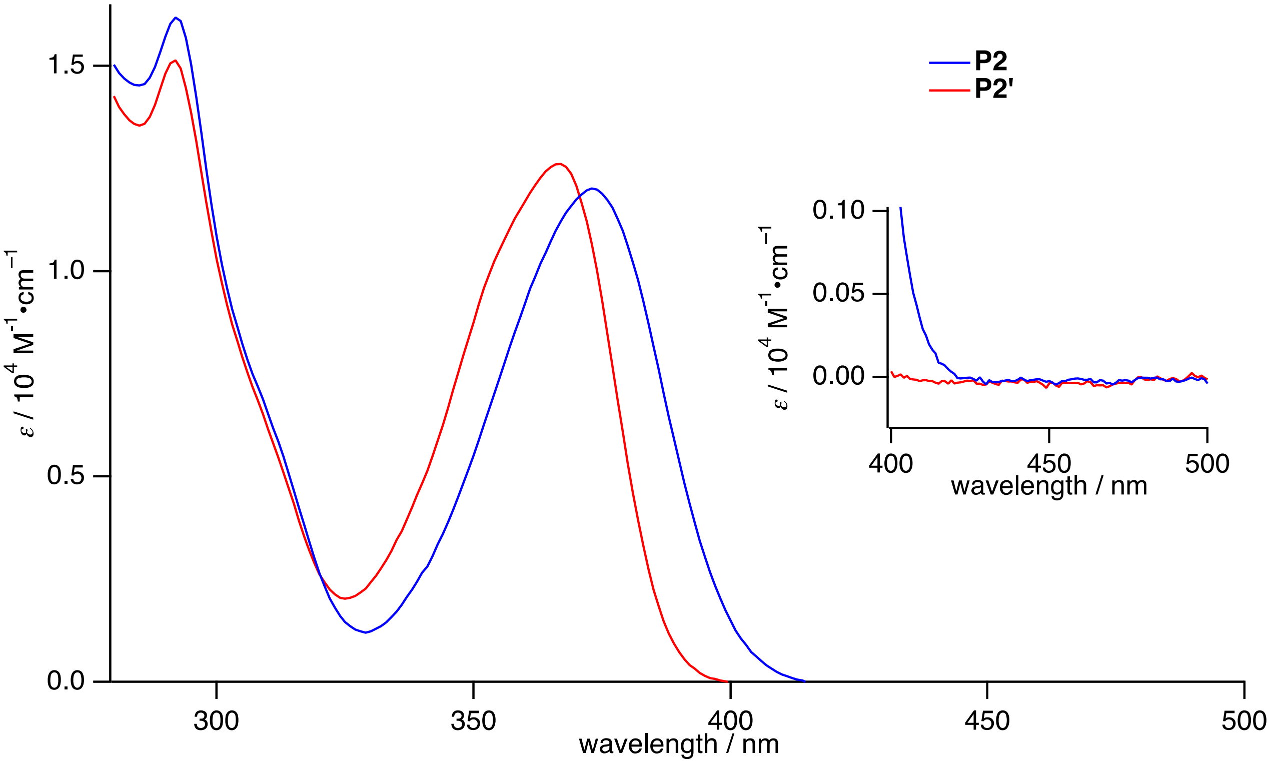

Figure S18. UV-vis absorption spectra of **P2** and **P2'** ($CHCl_3$, 10 μM).

(6) NMR spectral titration

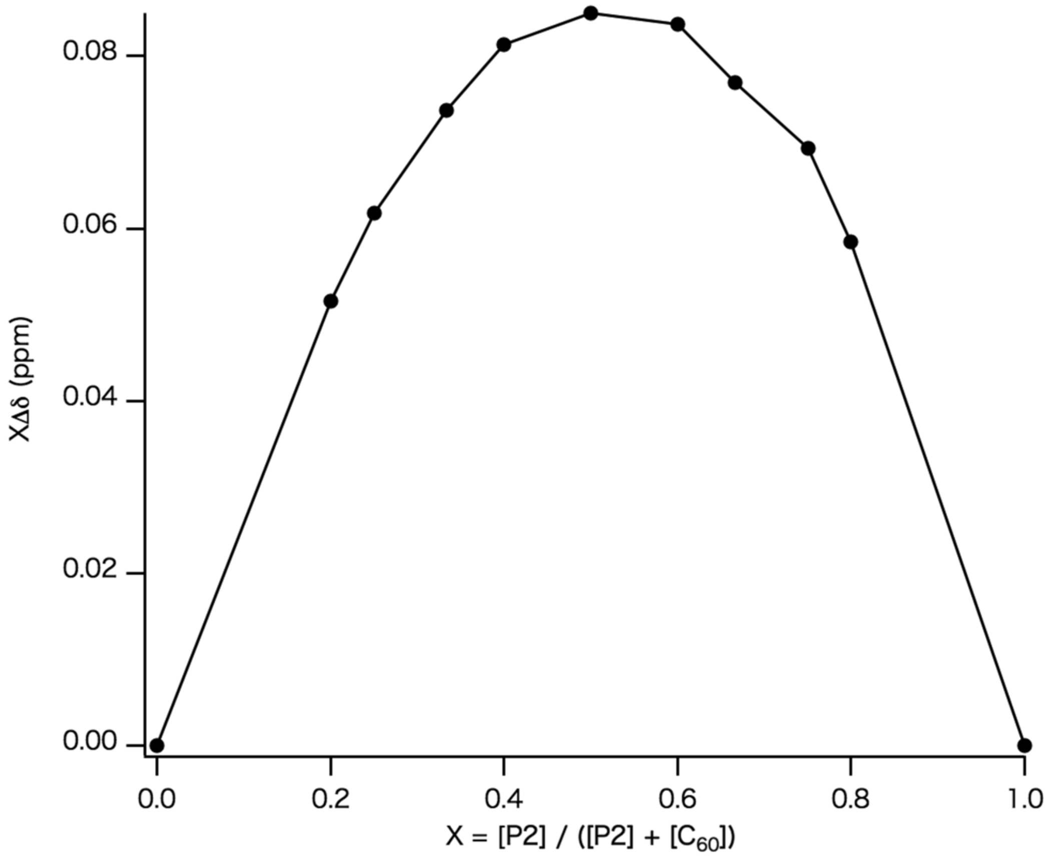

Figure S19. A Job plot for host-guest complexation of **P2** and C_{60} using ^1H NMR (600 MHz, $CDCl_3/CS_2$ (1:3 v/v), [**P2**] + [C_{60}] = 2.7 mM).

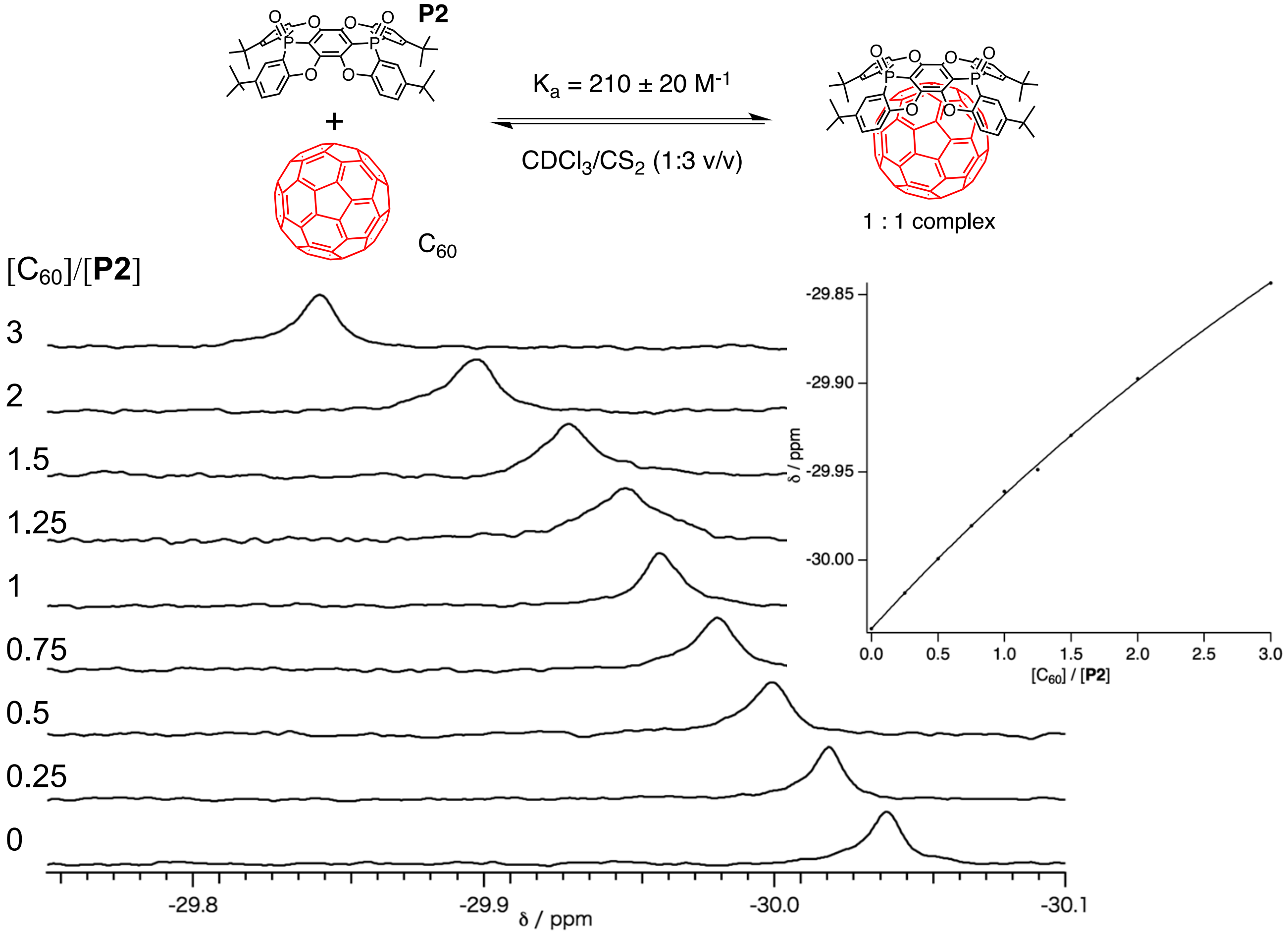

Figure S20. ^{31}P NMR spectral change of **P2** upon addition of C_{60} (242 MHz, $CDCl_3/CS_2$ (1:3 v/v), [**P2**] = 0.5 mM). Inset shows 1:1 binding isotherm.

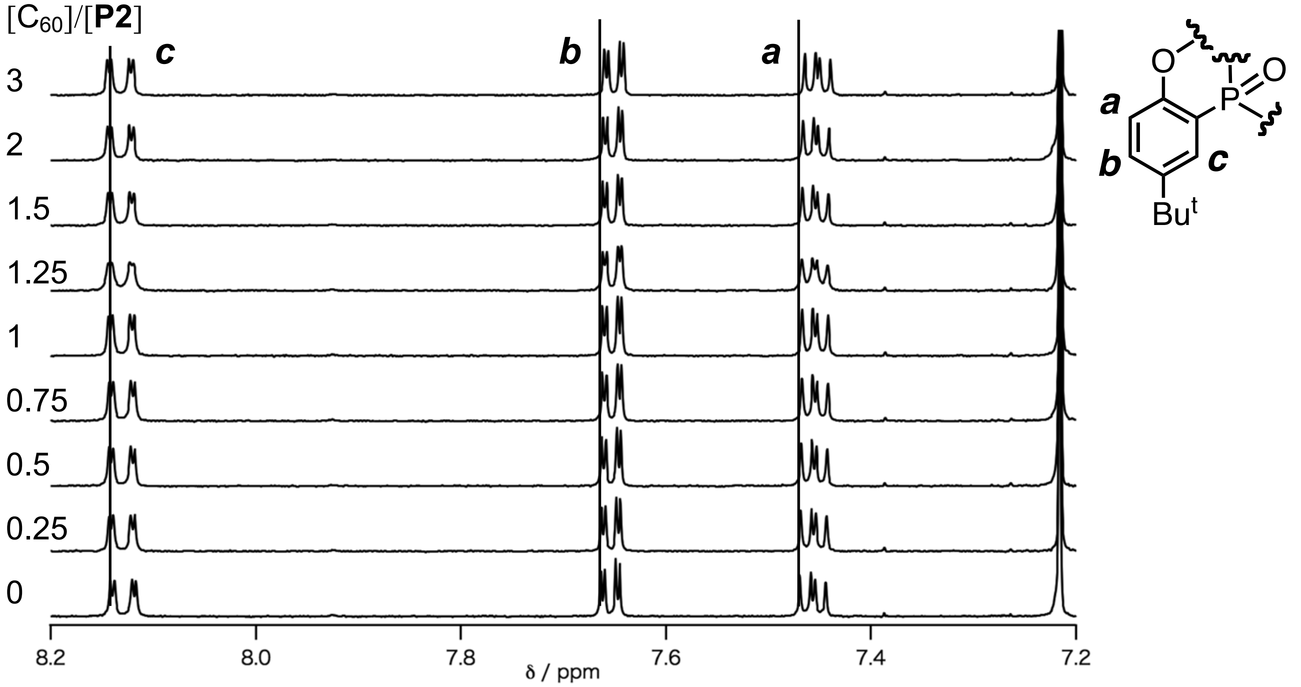

Figure S21. ^{1}H NMR spectral change of **P2** upon the addition of C_{60} (600 MHz, $CDCl_3/CS_2$ (1:3 v/v), [**P2**] = 0.5 mM). Dot lines are overlapped to emphasize chemical shift changes.

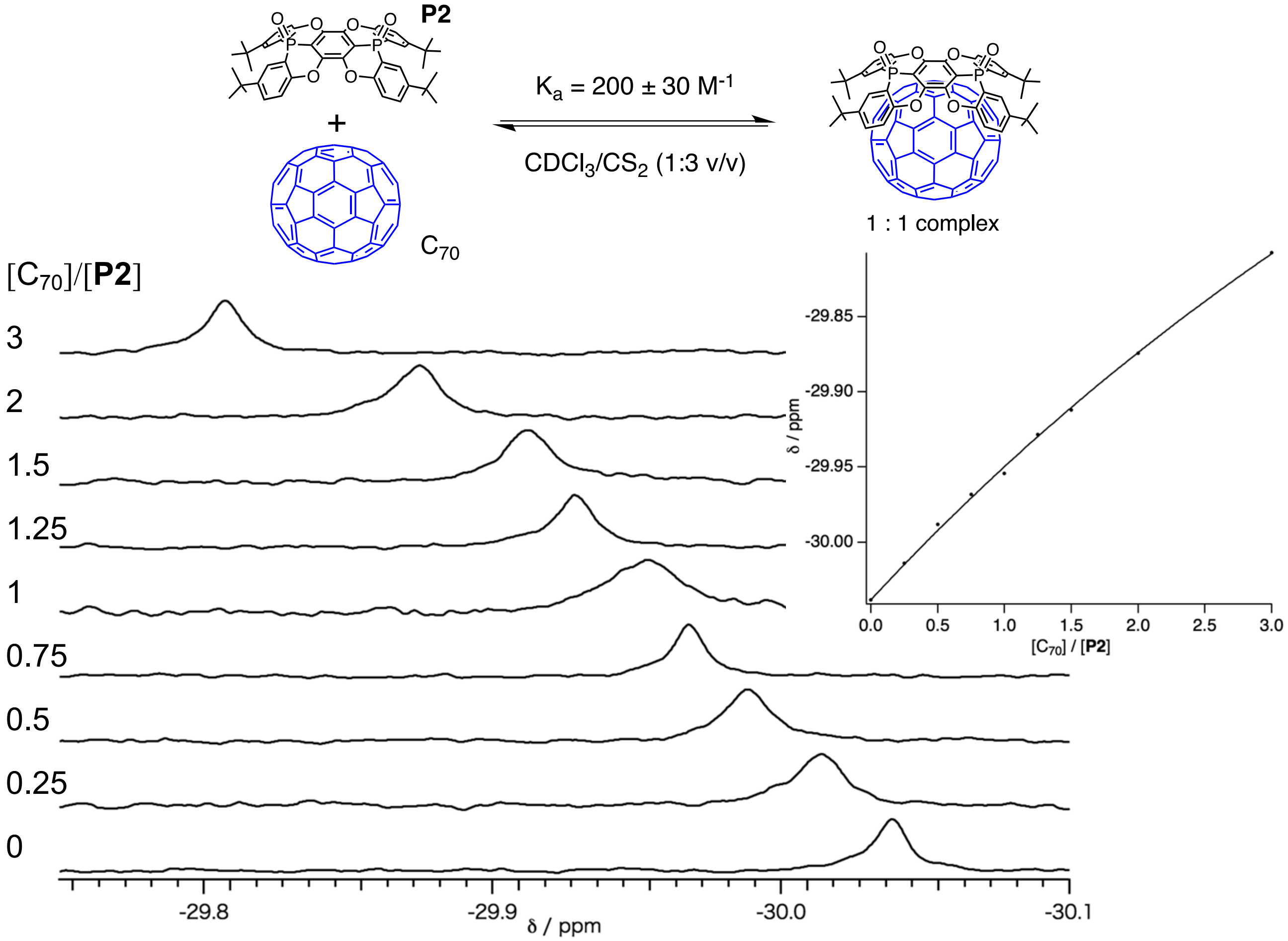

Figure S22. ^{31}P NMR spectral change of **P2** upon addition of C_{70} (242 MHz, $CDCl_3/CS_2$ (1:3 v/v), [**P2**] = 0.5 mM). Inset shows 1:1 binding isotherm.

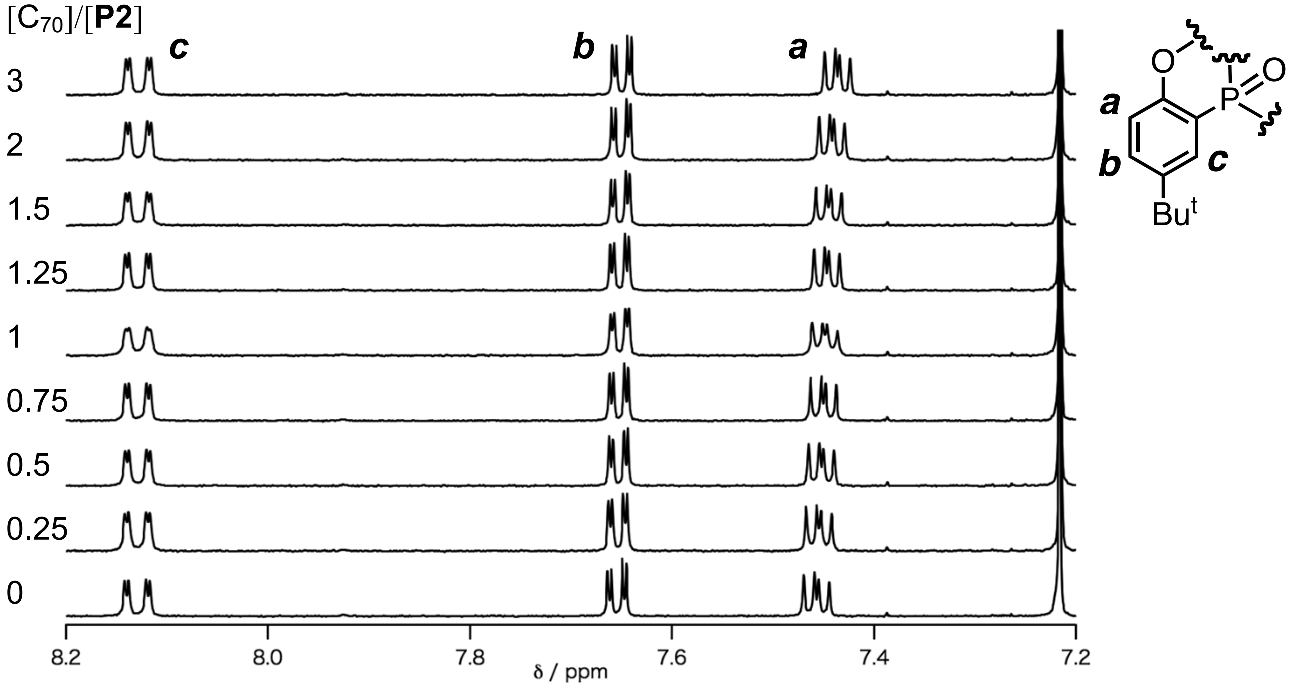

Figure S23. ^{1}H NMR spectral change of **P2** upon the addition of C_{70} (600 MHz, $CDCl_3/CS_2$ (1:3 v/v) , [**P2**] = 0.5 mM).

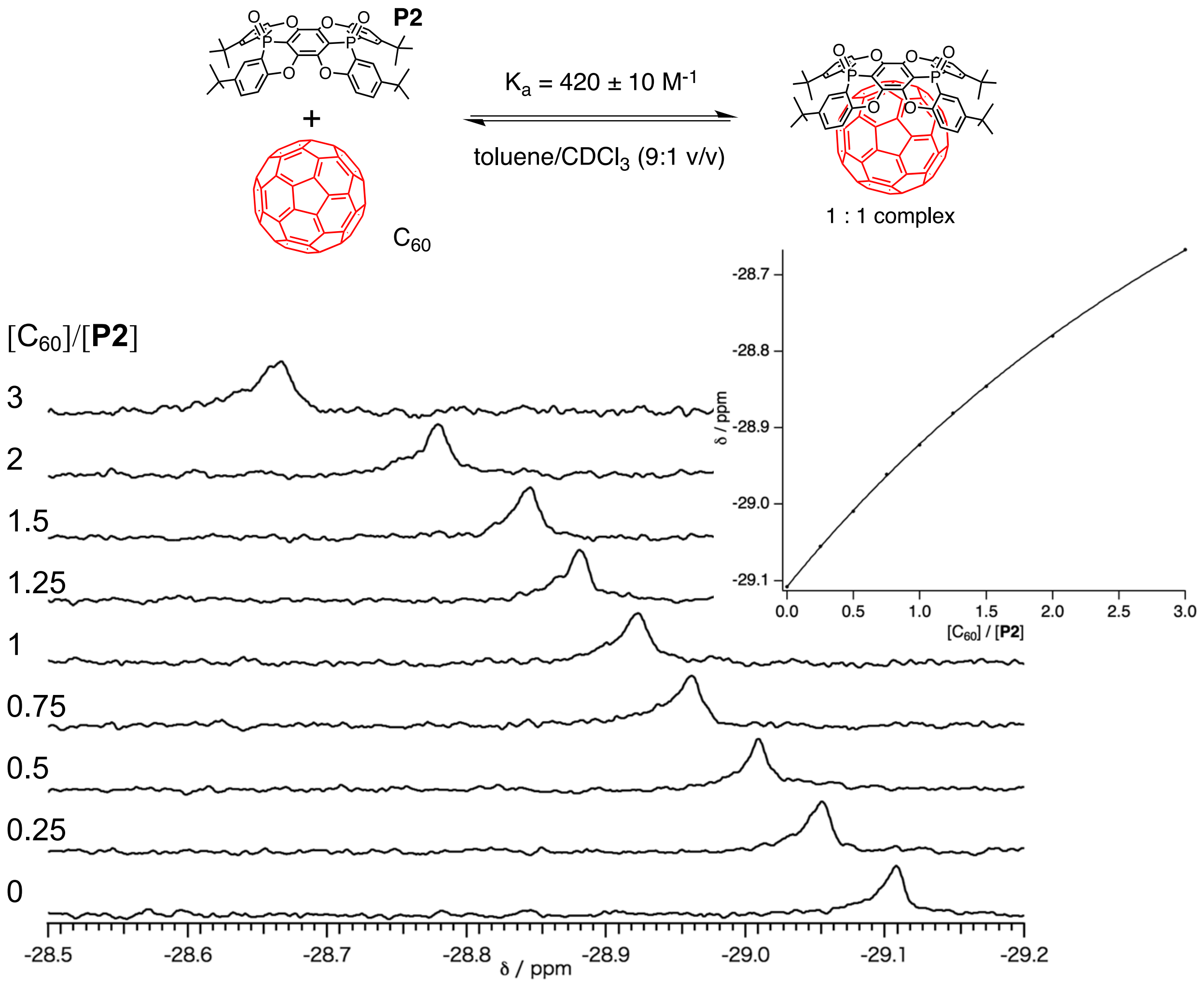

Figure S24. ^{31}P NMR spectral change of **P2** upon the addition of C_{60} (243 MHz, toluene/$CDCl_3$ (9:1 v/v), [**P2**] = 0.5 mM). Inset shows 1:1 binding isotherm.

(6) UV-vis absorption spectral titration

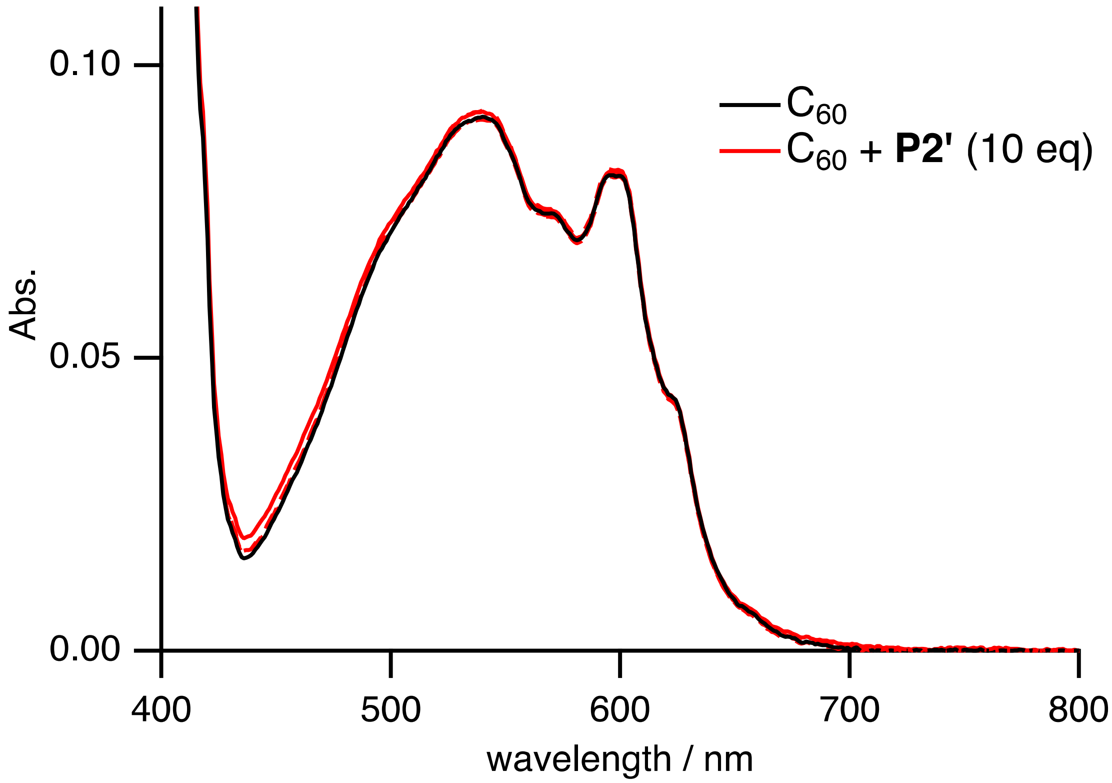

Figure S25. UV-vis spectral change of C_{60} upon the addition of **P2’** ($[C_{60}]$ = 0.1 mM, 0 ≤ [**P2’**]/$[C_{60}]$ ≤ 10, $CHCl_3$/toluene (1:4 v/v)).

(7) X-ray Crystallographic Analysis

X-ray diffraction measurements were performed using a Bruker APEXII ULTRA. The X-ray diffraction intensities were collected on a CCD diffractometer at 120 K using MoK□ (graphite-monochromated, λ = 0.71073 Å) radiation. The data were integrated with SAINT (Bruker, 2004), and an empirical absorption correction (SADABS) was applied. The structure was solved by the direct method of SHELXD-2014 and refined using the SHELXL-2014 program.[S2] All of the positional parameters and thermal parameters of non-hydrogen atoms were anisotropically refined on F^2 by the full-matrix least-squares method. Hydrogen atoms were placed at the calculated positions and refined riding on their corresponding carbon atoms. The crystallographic data for **P2**, **P2’**, (**P2**)$_2$⊃C_{60} (**I**), (**P2**)$_2$⊃C_{60} (**II**), (**P2**)$_2$⊃C_{60} (**III**), (**P2**)$_2$⊃C_{70} (**IV**), and (**P2**)$_2$⊃C_{70} (**V**) were deposited with the Cambridge Crystallographic Data Center as supplementary publications CCDC 1401373-1401379. Copies of the data can be obtained free of charge on application to CCDC, 12 Union Road, Cambridge CB2 1EZ, U.K. (fax: (+44) 1223-336-033; email: deposit@ccdc.cam.ac.uk).

A small depth *d* is defined as a distance between the centroids of the central benzene ring and the plane of the terminal carbons of the four benzene rings. A large depth *D* is defined as a distance between the centroids of the central benzene ring and the plane of the terminal carbons of the four *tert*-butyl groups.

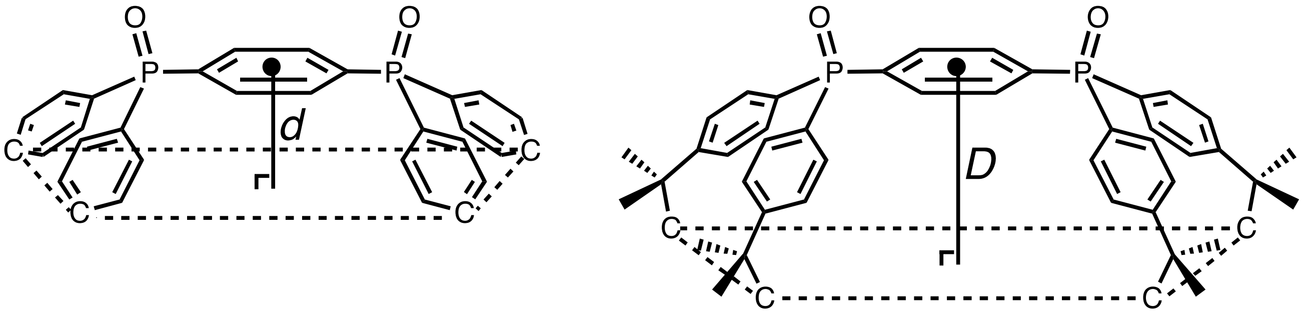

Figure S26. Definition of small depth *d* and large depth *D*.

Table S1. Crystallographic data.

	**P2** (*syn*)	**P2'** (*anti*)	(**P2**)$_2$⊃C_{60} (**I**)
Composite	$C_{46}H_{48}O_6P_2\cdot(CHCl_3)_4$	$C_{46}H_{48}O_6P_2\cdot(C_7H_8)_2$	$(C_{46}H_{48}O_6P_2)_2\cdot C_{60}$
Formula	$C_{50}H_{52}O_6P_2Cl_{12}$	$C_{60}H_{64}O_6P_2$	$C_{152}H_{96}O_{12}P_4$
Formula weight	1236.26	943.05	2238.16
Crystal size (mm^3)	0.60 × 0.40 × 0.10	0.30 × 0.05 × 0.05	0.20 × 0.05 × 0.05
Crystal system	monoclinic	triclinic	monoclinic
Space group	$P2_1/c$	P-1	$C2/m$
a (Å)	12.0579(14)	11.135(2)	34.540(2)
b (Å)	17.329(2)	11.644(3)	42.038(3)
c (Å)	27.445(3)	12.132(3)	17.4446(11)
α (deg)	90	97.161(2)	90
β (deg)	91.2630(10)	111.866(2)	100.248(3)
γ (deg)	90	112.432(2)	90
V (Å^3)	5733.4(12)	1282.4(5)	24925(3)
Z	4	1	8
D_{calcd} (g·cm^{-3})	1.432	1.221	1.193
Collected/Unique	32282/13151	32000/6222	439608/ 46996
R_{int}	0.0228	0.0431	0.0749
θ_{max} (deg)	27.59	28.22	25.24
F_{000}	2536	502	9312
μ (MoKα) (mm^{-1})	0.681	0.136	0.312
Limiting indices	$-15 \le h \le 14$	$-14 \le h \le 14$	$-53 \le h \le 52$
	$-21 \le k \le 22$	$-15 \le k \le 15$	$-63 \le k \le 63$
	$-35 \le l \le 32$	$-16 \le l \le 16$	$-26 \le l \le 26$
Parameters/restraints	697/0	314/0	1450/127
Goodness of fit (F^2)	1.057	1.019	1.233
R_1 ($I > 2\sigma(I)$)	0.0421	0.0447	0.1314
wR_2 (all date)	0.1097	0.1149	0.3117
Largest peak (e·Å^{-3})	0.631	0.559	1.758
Largest hole (e·Å^{-3})	–0.480	–0.416	–2.258

$R_1 = \sum||F_o|-|F_c|/\sum|F_o|$, $wR_2 = \{\sum[w(F_o^2-F_c^2)^2]/\sum[w(F_o^2)^2]\}^{1/2}$

	$(\mathbf{P2})_2{\supset}C_{60}$ (**II**)	$(\mathbf{P2})_2{\supset}C_{60}$(**III**)	$(\mathbf{P2})_2{\supset}C_{70}$ (**IV**)
Composite	$(C_{46}H_{48}O_6P_2)_2{\cdot}C_{60}$	$(C_{46}H_{48}O_6P_2)_2{\cdot}C_{60}{\cdot}$ $(CHCl_3)_4$	$(C_{46}H_{48}O_6P_2)_2{\cdot}C_{70}{\cdot}$ $(CHCl_3)_2$
Formula	$C_{152}H_{96}O_{12}P_4$	$C_{156}H_{100}O_{12}P_4Cl_{12}$	$C_{164}H_{98}O_{12}P_4Cl_6$
Formula weight	2238.16	2715.64	2597.0
Crystal size (mm^3)	0.10 × 0.05 × 0.05	0.15 × 0.10 × 0.05	0.30 × 0.15 × 0.03
Crystal system	monoclinic	monoclinic	orthorhombic
Space group	$P2_1/c$	$C2/c$	$P2_12_12$
a (Å)	24.148(8)	25.827(5)	21.980(5)
b (Å)	23.858(8)	22.684(4)	17.101(4)
c (Å)	22.983(8)	22.259(4)	17.472(4)
α (deg)	90	90	90
β (deg)	117.759(4)	99.466(2)	90
γ (deg)	90	90	90
V (Å^3)	11717(7)	12863(4)	6567(2)
Z	4	4	2
D_{calcd} ($g{\cdot}cm^{-3}$)	1.269	1.402	1.313
Collected/Unique	255517/28158	36501/15982	41016/12313
R_{int}	0.0917	0.0205	0.0485
θ_{max} (deg)	25.242	25.242	25.242
F_{000}	4656	5584	2680
μ (MoKα) (mm^{-1})	0.131	0.374	0.245
Limiting indices	$-31 \le h \le 31$	$-34 \le h \le 33$	$-26 \le h \le 24$
	$-31 \le k \le 31$	$-15 \le k \le 29$	$-20 \le k \le 20$
	$-30 \le l \le 30$	$-27 \le l \le 29$	$-21 \le l \le 21$
Parameters/restraints	1581/87	868/69	880/63
Goodness of fit (F^2)	1.293	1.365	1.073
R_1 ($I > 2\sigma(I)$)	0.1420	0.1035	0.0580
wR_2 (all date)	0.3457	0.2534	0.1303
Largest peak (e·Å^{-3})	1.276	1.264	0.306
Largest hole (e·Å^{-3})	–0.743	–0.986	–0.513
Flack			0.03(3)

$R_1 = \sum||F_o|-|F_c|/\sum|F_o|$, $wR_2 = \{\sum[w(F_o^2-F_c^2)^2]/\sum[w(F_o^2)^2]\}^{1/2}$

	($\mathbf{P2}$)$_2$⊃C_{70} (**V**)
Composite	$(C_{46}H_{48}O_6P_2)_2\cdot C_{70}\cdot (CHCl_3)_4$
Formula	$C_{166}H_{100}O_{12}P_4Cl_{12}$
Formula weight	2835.7
Crystal size (mm^3)	0.25 × 0.05 × 0.05
Crystal system	monoclinic
Space group	*C*2/*c*
a (Å)	34.079(12)
b (Å)	13.764(5)
c (Å)	27.024(10)
α (deg)	90
β (deg)	103.463(4)
γ (deg)	90
V (Å^3)	12328(8)
Z	4
D_{calcd} (g·cm^{-3})	1.528
Collected/Unique	45630/8926
R_{int}	0.1044
θ_{max} (deg)	25.242
F_{000}	5824
μ (MoKα) (mm^{-1})	0.394
Limiting indices	$-36 \le h \le 37$
	$-15 \le k \le 14$
	$-29 \le l \le 29$
Parameters/restraints	886/12
Goodness of fit (F^2)	1.416
R_1 ($I > 2\sigma(I)$)	0.1447
wR_2 (all date)	0.3803
Largest peak (e·Å^{-3})	1.128
Largest hole (e·Å^{-3})	–1.760

$R_1 = \sum||F_o|-|F_c|/\sum|F_o|$, $wR_2 = \{\sum[w(F_o^2-F_c^2)^2]/\sum[w(F_o^2)^2]\}^{1/2}$

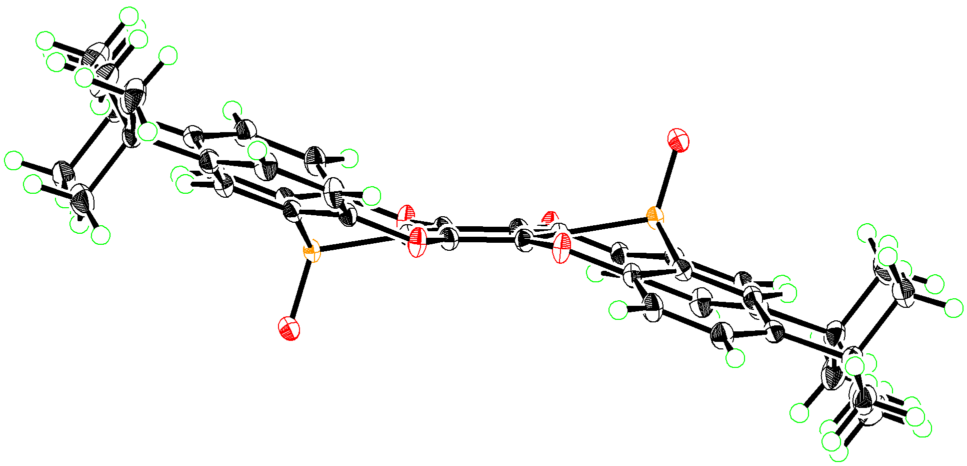

Figure S27. *ORTEP* drawing of **P2'**; top (left) and side (right) views; thermal ellipsoids set at 50% probability.

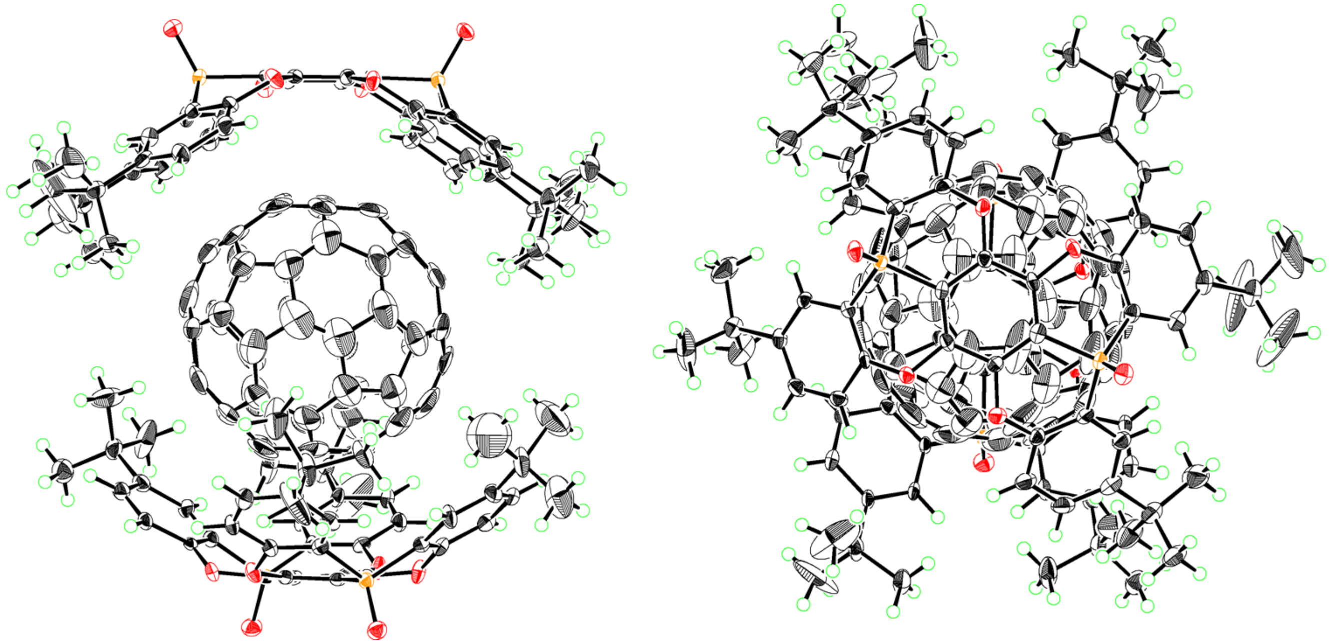

Figure S28. *ORTEP* drawing of **(P2)**$_2 \supset C_{60}$ **(I)**; side (left) and top (right) views; thermal ellipsoids set at 50% probability.

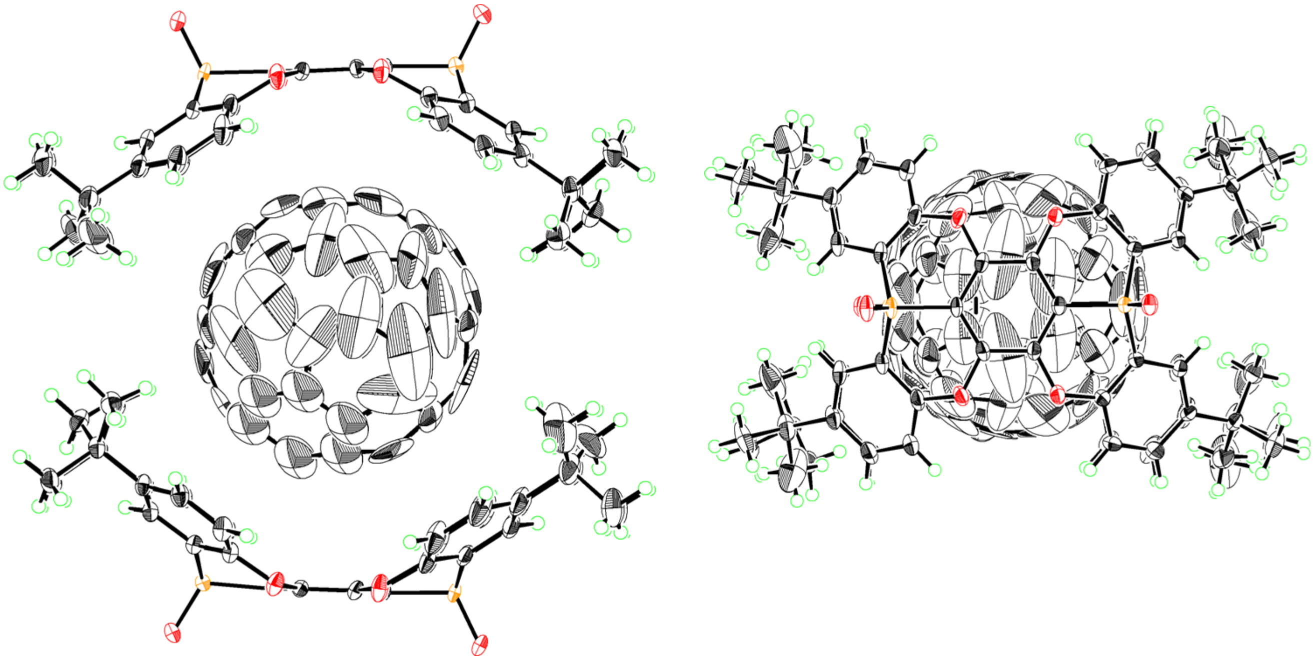

Figure S29. *ORTEP* drawing of one of the two independent molecules of (**P2**)$_2$⊃C_{60} (**II**); side (left) and top (right) views; thermal ellipsoids set at 50% probability.

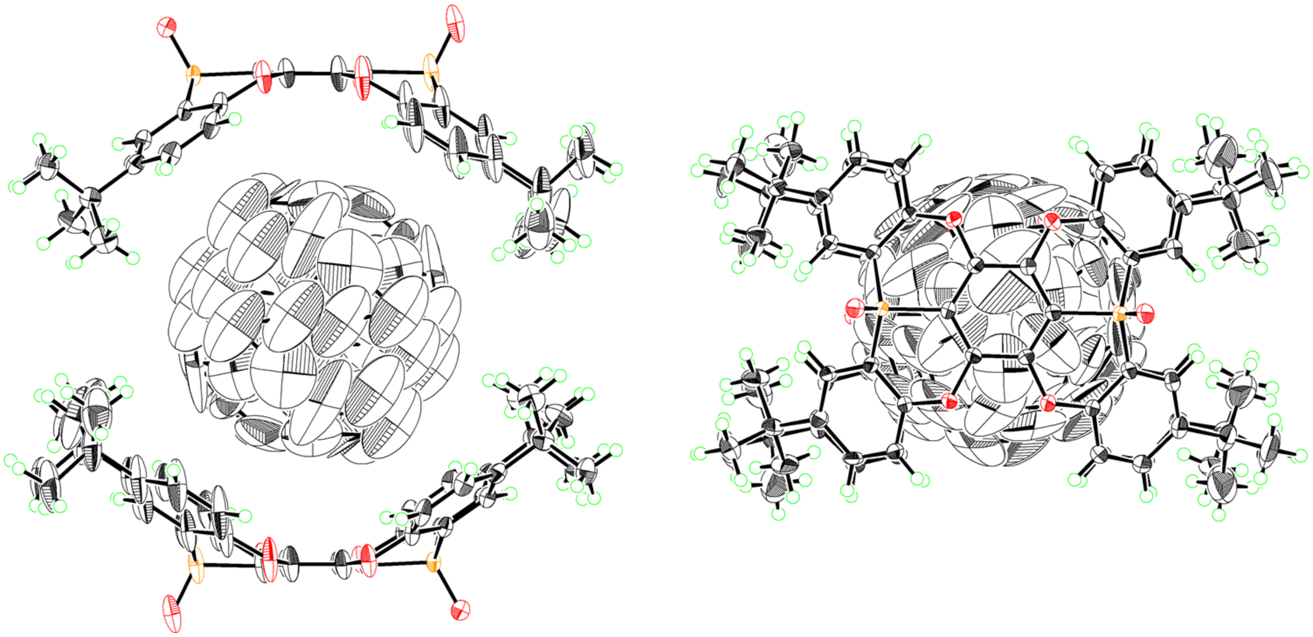

Figure S30 *ORTEP* drawing of the other of the two independent molecules of (**P2**)$_2$⊃C_{60} (**II**) side (left) and top (right) views; thermal ellipsoids set at 50% probability.

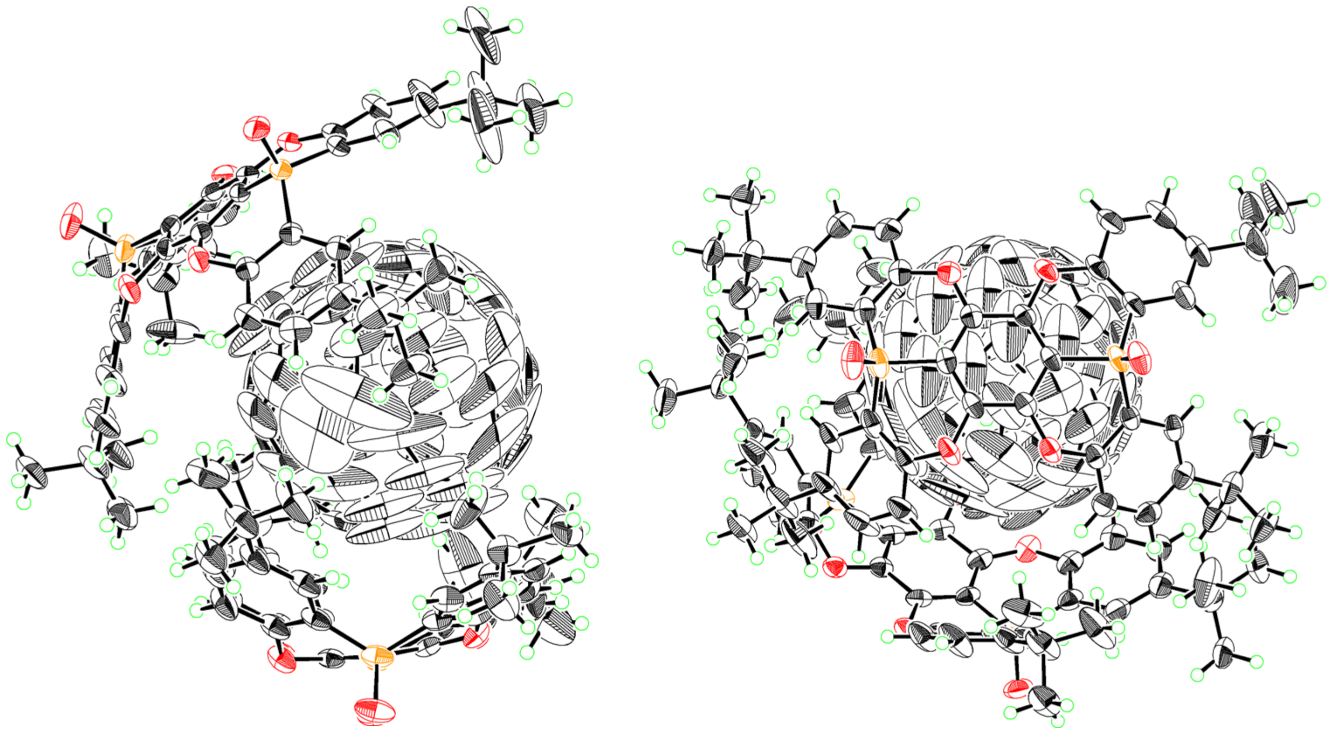

Figure S31. *ORTEP* drawing of (**P2**)$_2$⊃C_{60} (**III**), top (left) and side (right) views; thermal ellipsoids set at 50% probability. Solvent molecules are omitted for clarity.

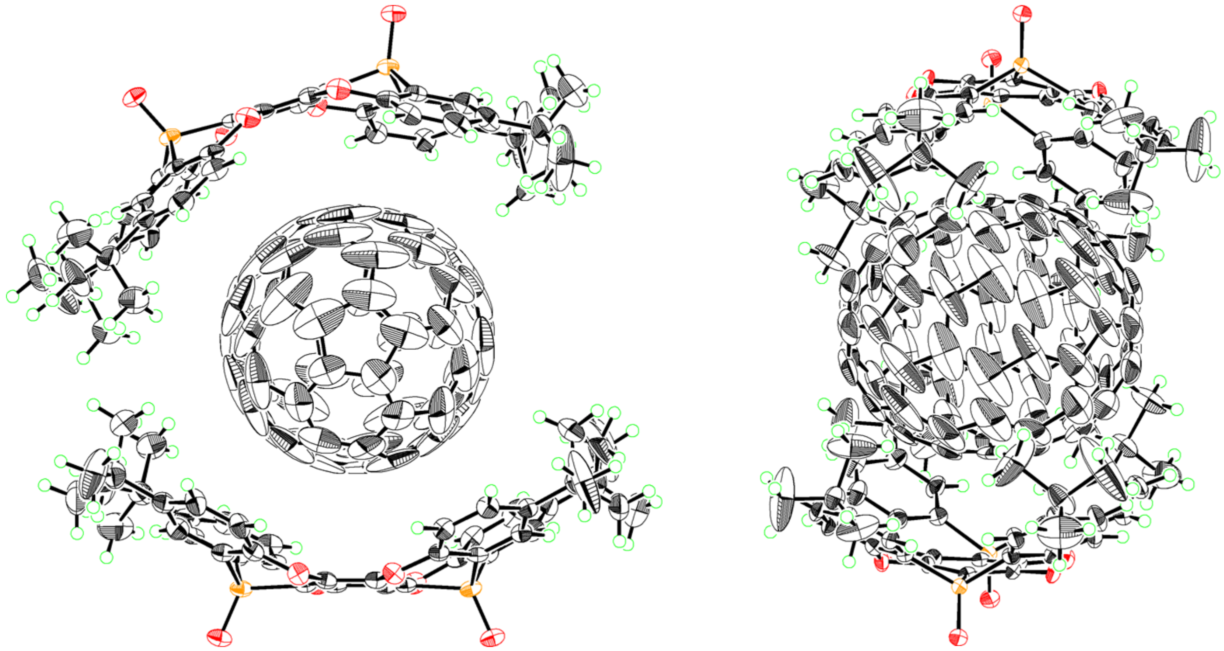

Figure S32. *ORTEP* drawing of (**P2**)$_2$⊃C_{70} (**IV**), top (left) and side (right) views; thermal ellipsoids set at 50% probability. Solvent molecules are omitted for clarity.

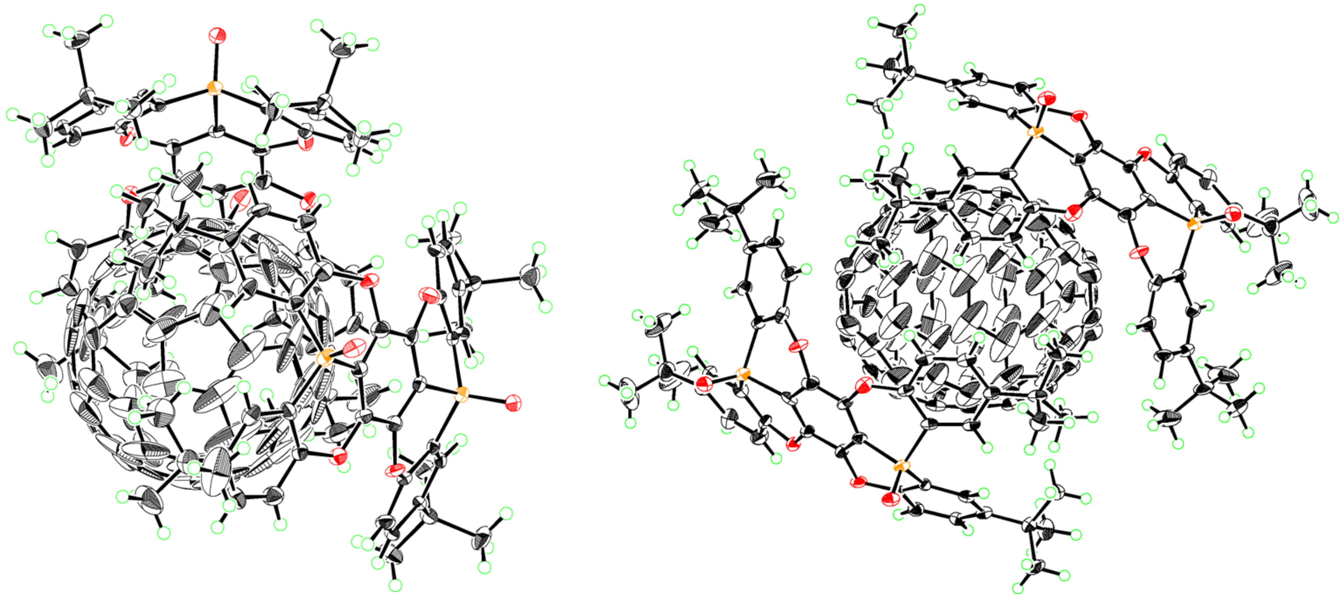

Figure S33. *ORTEP* drawing of (**P2**)$_2$⊃C_{70} (**V**), top (left) and side (right) views; thermal ellipsoids set at 50% probability. Solvent molecules are omitted for clarity.

(8) References

[S1] T. Senda, M. Ogasawara, T. Hayashi, *J. Org. Chem.* 2001, **66**, 6852-6856.

[S2] (a) G. M. Sheldrick, *Acta Crystallogr. Sect. A* 2008, **64**, 112-122. (b) G. M. Sheldrick, *SHELX-2014/6, Program for crystal structure determination*; Universität Göttingen: Göttingen, Germany, 2014.
